# Initial levels of β-amyloid and tau deposition have distinct effects on longitudinal tau accumulation in Alzheimer’s disease

**DOI:** 10.1186/s13195-023-01178-w

**Published:** 2023-02-07

**Authors:** Yue Cai, Jing Du, Anqi Li, Yalin Zhu, Linsen Xu, Kun Sun, Shaohua Ma, Tengfei Guo, for the Alzheimer’s Disease Neuroimaging Initiative

**Affiliations:** 1 Institute of Biomedical Engineering, Shenzhen Bay Laboratory, No.5 Kelian Road, Shenzhen, 518132 China; 2 Tsinghua Shenzhen International Graduate School (SIGS), Tsinghua University, Shenzhen, 518055 China; 3 Department of Medical Imaging, University of Chinese Academy of Sciences-Shenzhen Hospital, Shenzhen, 518106 China; 4 Institute of Cancer Research, Shenzhen Bay Laboratory, Shenzhen, 518132 China; 5 Institute of Biomedical Engineering, Peking University Shenzhen Graduate School, Shenzhen, 518055 China

**Keywords:** Alzheimer’s disease, PET imaging, β-Amyloid, Tau, Longitudinal

## Abstract

**Background:**

To better assist with the design of future clinical trials for Alzheimer’s disease (AD) and aid in our understanding of the disease’s symptomatology, it is essential to clarify what roles β-amyloid (Aβ) plaques and tau tangles play in longitudinal tau accumulation inside and outside the medial temporal lobe (MTL) as well as how age, sex, apolipoprotein E (APOE) ε4 (APOE-ε4), and Klotho-VS heterozygosity (KL-VS^het^) modulate these relationships.

**Methods:**

We divided the 325 Aβ PET-positive (A+) participants into two groups, A+/T− (*N* = 143) and A+/T+ (*N* = 182), based on the threshold (1.25) of the temporal meta-ROI 18F-flortaucipir (FTP) standardized uptake value ratio (SUVR). We then compared the baseline and slopes of A+/T− and A+/T+ individuals’ Aβ plaques and temporal meta-ROI tau tangles with those of A−/T− cognitively unimpaired individuals (*N* = 162) without neurodegeneration. In addition, we looked into how baseline Aβ and tau may predict longitudinal tau increases and how age, sex, APOE-ε4, and KL-VS^het^ affect these associations.

**Results:**

In entorhinal, amygdala, and parahippocampal (early tau-deposited regions of temporal meta-ROI), we found that baseline Aβ and tau deposition were positively linked to more rapid tau increases in A+/T− participants. However, in A+/T+ individuals, the longitudinal tau accumulation in fusiform, inferior temporal, and middle temporal cortices (late tau-deposited regions of temporal meta-ROI) was primarily predicted by the level of tau tangles. Furthermore, compared to older participants (age ≥ 65), younger individuals (age < 65) exhibited faster Aβ-dependent but slower tau-related tau accumulations. Additionally, compared to the KL-VS^het−^ group, KL-VS^het+^ individuals showed a significantly lower rate of tau accumulation associated with baseline entorhinal tau in fusiform and inferior temporal regions.

**Conclusion:**

These findings offer novel perspectives to the design of AD clinical trials and aid in understanding the tau accumulation inside and outside MTL in AD. In particular, decreasing Aβ plaques might be adequate for A+/T− persons but may not be sufficient for A+/T+ individuals in preventing tau propagation and subsequent downstream pathological changes associated with tau.

**Supplementary Information:**

The online version contains supplementary material available at 10.1186/s13195-023-01178-w.

## Background

Extracellular β-amyloid (Aβ) plaques and intracellular neurofibrillary tau tangles are two main features of Alzheimer’s disease (AD) [1] that can be detected by positron emission tomography (PET) imaging in live brain tissues [2]. Aβ plaques and tau tangles detected by PET have demonstrated strong concordance with post-mortem findings in AD [3, 4]. Recent research results reached by our group [5] and other labs [6, 7] revealed that Aβ pathology is linked to tau increases in AD [8–11], and rather than Aβ plaques, aberrant neocortical tau aggregations are more strongly associated with neurodegeneration and cognitive decline in AD [5, 6, 12].

Previous post-mortem [13] and neuroimaging [14, 15] studies have shown that tau tangles typically spread systematically across the medial temporal lobe (MTL, Braak stages I–III), encompassing hippocampus, transentorhinal, and parahippocampal cortices [16], followed by their expansion to the association cortices (Braak stages III–IV), and eventually reaching the isocortical regions (Braak stages V–VI) in amnestic/typical AD [17, 18]. The initial tau aggregations can be observed in the entorhinal cortex of Aβ-negative people [11, 19], but those aggregations seldom spread outside of the entorhinal cortex unless there is a significant amount of neocortical Aβ plaques [11, 20–23]. Existing tau tangles [11, 24–26] and baseline Aβ plaques [8–11] can both indicate a faster tau accumulation in AD. However, it is still unclear how those two indicators impact the longitudinal accumulation in MTL and tau propagation from MTL to inferior/middle temporal cortices in various A/T profiles defined by the abnormal status of Aβ PET (A±) and tau PET (T±) [5]. Thus, examining factors influencing tau tangle propagation inside and outside of MTL in various A/T profiles may offer novel perspectives on comprehending the characteristics of tau pathology and formulating anti-AD clinical trial designs. Additionally, it has been suggested that APOE-ε4 [11, 27], Klotho-VS heterozygosity (KL-VS^het^) [28], sex [29, 30], and age [20, 22, 24, 26, 29] may all be involved in tau increases. Further investigation is therefore necessary for determining how those factors interact with baseline Aβ plaques and tau levels in predicting longitudinal tau accumulation in early Braak stages.

In this study, we examined the PET images of participants from the Alzheimer’s Disease Neuroimaging Initiative (ADNI) cohort to investigate how baseline Aβ and tau levels affect longitudinal tau propagation over time in MTL, inferior temporal, and middle temporal cortices, as well as how age, sex, APOE-ε4, and KL-VS^het^ modulate these relations. The ultimate goal is to comprehend what contributes to the longitudinal tau accumulation in cortical regions at early Braak stages (I–IV) in AD.

## Methods

### Participants

Data were obtained from the ADNI database (ida.loni.usc.edu). The ADNI was launched in 2003 as a public-private partnership, led by principal investigator Michael W. Weiner, MD. The primary goal of ADNI has been to examine whether serial magnetic resonance imaging (MRI), PET, other biological markers, and clinical and neuropsychological assessments can be combined to evaluate the progression of mild cognitive impairment (MCI) and early AD. The ADNI study was approved by the institutional review boards of all participating centers, and written informed consent was obtained from all participants or their authorized representatives.

Using concurrent (acquisition interval within 1 year) Aβ PET (^18^F-florbetapir (FBP) or ^18^F-florbetaben (FBB)), tau PET (^18^F-flortaucipir (FTP)) and anatomical MRI, we identified 141 cognitively unimpaired (CU) participants, 119 MCI participants, and 65 AD patients who were Aβ PET positive (A+) at baseline. For the control group, we included 162 CU participants who were Aβ PET negative (A−) and tau PET negative (T−) and did not exhibit any evidence of hippocampal atrophy or cortical thickness shrinking (calculation and thresholds attached in Supplementary material). Longitudinally, 192 individuals had at least one follow-up (FU) FTP PET scan.

### PET imaging processing

Details about FBP, FBB, and FTP image acquisition and analysis can be found at http://adni-info.org. Briefly, PET data were acquired in 5-min frames from 50 to 70 min (FBP), 90 to 110 min (FBB), and 75 to 105 min (FTP) post-injection. The fully preprocessed FBP PET, FBB PET, FTP PET, and T1-weighted anatomical MRI images were downloaded from the LONI website (ida.loni.usc.edu). Baseline and follow-up FBP, FBB, and FTP scans were coregistered to their anatomical MRI scans that were closest in time to PET scans. FreeSurfer V5.3.0 extracted cortical Aβ tracer retention in 68 regions of interest (ROIs) defined by the Desikan-Killiany atlas [31] based on the coregistered PET images in individual MRI space. FBP or FBB standardized uptake value ratios (SUVRs) were obtained by normalizing regional FBP or FBB uptake to that in the whole cerebellum reference region. The AD summary cortical SUVR was calculated as a volume-weighted SUVR of frontal, cingulate, parietal, and lateral temporal regions; A+ was defined by AD summary cortical SUVR of FBP ≥ 1.11 or FBB ≥ 1.08, and FBP and FBB SUVRs were converted to Centiloids as described previously [32].

FTP uptakes in 68 cortical ROIs defined by the Desikan-Killiany atlas [31] and one composite temporal meta-ROI [33] were extracted in the native MRI space. Temporal meta-ROI is a composite ROI comprising entorhinal, amygdala, parahippocampal, fusiform, inferior temporal, and middle temporal regions. Regional FTP SUVRs were calculated using inferior cerebellum intensity normalization [33] and the threshold of temporal meta-ROI FTP SUVR was set as ≥ 1.25 as described previously [25]. For the sensitivity analysis, we used an alternative threshold of temporal meta-ROI FTP SUVR (≥1.27) defined by another independent cohort [34] to describe T±. As suggested by conclusions yielded from previous studies [25, 35] that white matter intensity normalization has shown more statistical power to detect longitudinal tau PET changes [25, 35], FTP slopes were calculated using the white matter reference region.

### Definition of subgroups

A+ participants were further divided into A+/T− and A+/T+ groups according to the threshold of temporal meta-ROI FTP SUVR described above. The cohort was separated into subgroups to explore how age, sex, APOE-ε4, and KL-VS^het^ affect longitudinal tau accumulation. Sixty-five was selected as the cut-off age to divide the entire cohort into early-life and late-life elderly adults [36]. For the sensitivity analysis, we used all participants’ median age (72.7 years) to define early-life and late-life elderly adults. Two single-nucleotide polymorphisms for KL-VS^het^ (rs9536314 for F352V, rs9527025 for C370S) and APOE (rs429358, rs7412) were genotyped using DNA extracted by Cogenics from a 3-mL aliquot of EDTA blood as described in ADNI website (see ida.loni.usc.edu). Participants with one but not two copies of the Klotho-VS haplotype [28] were defined as the KL-VS^het+^ group, and participants without any Klotho-VS haplotype as the KL-VS^het−^ group. Besides, participants who carried at least one ε4 allele were defined as APOE-ε4 carriers and the rest as APOE-ε4 non-carriers.

### Statistical analysis

All statistical analyses were performed using statistical program R (v4.0.4, The R Foundation for Statistical Computing). Demographical data were presented as median (interquartile range (IQR)) for continuous characteristics and percentage (%) for discrete characteristics. Demographical characteristics at baseline among the control, A+/T−, and A+/T+ groups were compared using a two-tailed Mann-Whitney *U* test or Fisher’s exact test. A false discovery rate (FDR) of 0.05 was applied using the Benjamini-Hochberg approach for multiple comparison correction. Linear mixed effect models were used to calculate slopes of Centiloids (∆ Aβ Centiloids) and FTP SUVR (∆ FTP SUVR) over time based upon the longitudinal data, including time, age at baseline, and sex as independent variables, and a random slope and intercept for each individual.

Generalized linear models (GLM) were used to compare baseline and slopes of Aβ Centiloids and FTP SUVRs in temporal meta-ROI and individual ROIs within the composite region among the control, A+/T−, and A+/T+ groups, controlling for age and sex. Furthermore, the sequential tau propagation order was evaluated by symmetric matrices of baseline and slopes of FTP SUVRs among 6 ROIs within the temporal meta-ROI, in which the element of symmetric matrices indicated the strength of correlations among ROIs. Interregional correlation coefficients were calculated using Pearson partial correlation (R; Ggm package) across the whole cohort and were adjusted for age, sex, and APOE-ε4 as well as baseline FTP SUVRs or ∆ FTP SUVRs of all remaining ROIs; Bonferroni-corrected for multiple comparisons was set as *p* < 0.05.

We used model 1 to investigate how global cortical Aβ burden and initial tau deposition relate to further tau aggregation in 6 ROIs of the temporal meta-ROI in A+/T− and A+/T+ participants separately. To avoid losing crucial information about the continuous relation between Aβ PET and longitudinal tau PET, we repeated the analysis using the continuous measure of Aβ Centiloids in the whole cohort and in T− (combining A−/T−/N−/CU and A+/T−) and T+ (A+/T+) subgroups.

Model 1: *Slope of tau PET ~ Aβ PET + tau PET + age + sex*

To investigate how age, sex, APOE-ε4, and KL-VS^het^ influence longitudinal tau aggregation, we used models 2 and 3 to determine how each factor interacted with baseline Aβ and tau in predicting longitudinal tau propagation over time.

Model 2: *Slope of tau PET ~ Aβ PET × factor*

Model 3: *Slope of tau PET ~ Aβ PET + tau PET × factor*

In the models above, “factor” referred to the subgroups of age, sex, APOE-ε4, or KL-VS^het^ genotyping, respectively. Considering that KL-VS^het+^ is associated with reduced Aβ deposition and cognitive decline in APOE-ε4 carriers, as reported previously [37], APOE-ε4 was also included as a covariate in all KL-VS^het^-associated models. We compared the slopes of tau PET in temporal meta-ROI and 6 ROIs within this composite region between KL-VS^het−^ individuals and KL-VS^het+^ individuals. The difference in slope was quantitatively assessed by a KL-VS^het+^/ KL-VS^het−^ ratio, calculated by the median slope of tau accumulation in the KL-VS^het+^ group divided by the median slope in the KL-VS^het−^ group.

Mediation analysis was then performed using latent variable modeling to elucidate further the associations among KL-VS^het^ genotyping, baseline global Aβ burden, initial tau tangle, and longitudinal tau propagation (R; Lavaan package) to figure out how KL-VS^het^ modulated longitudinal tau increases. All variables were converted to standard *z*-scores. Total, direct, and indirect effects were calculated via a 5000-iteration bootstrapping procedure.

Finally, we did a sensitivity analysis to compare baseline and longitudinal FTP SUVRs among the control, A+/T−, and A+/T+ groups and to predict the longitudinal changes of FTP SUVRs in A+/T− and A+/T+ groups using the alternative threshold of temporal meta-ROI FTP SUVR (≥1.27).

## Results

### Demographics

The demographical characteristics of participants in this study can be found in Table 1. At baseline, compared to those in the control group, A+/T− and A+/T+ individuals had older ages (estimate = 4.942, [95% confidence interval (ci), 3.343~6.563], *p* < 0.001; estimate = 5.407, [95% ci, 3.934~6.962], *p* < 0.001) and a higher percentage of APOE-ε4 carriers (odds ratio = 2.848, [95% ci, 1.708~4.794], *p* < 0.001; odds ratio = 5.992, [95% ci, 3.658~9.965], *p* < 0.001). A+/T+ individuals had a shorter duration of education than both the A+/T− group (estimate = −1.000, [95% ci, −2.000~−0.00006], *p* = 0.008) and the control group (estimate = −1.000, [95% ci, −2.000~−0.00002], *p* = 0.001). A+/T+ also had a higher percentage of APOE-ε4 carriers (odds ratio = 2.104, [95% ci, 1.294 ~ 3.439], *p* = 0.002) than the A+/T− groups. In addition, we found that A+/T− individuals had fewer females (odds ratio = −0.530, [95% ci, 0.329~0.850], *p* = 0.006) than the control group. No other difference was found. Longitudinally, 199 and 192 individuals had at least two Aβ and FTP PET scans, respectively. More detailed demographics of individuals with longitudinal Aβ PET and tau PET can be found in Table 1. Additionally, the demographical characteristics of participants in different groups defined by alternative FTP SUVR threshold of temporal meta-ROI (1.27) can be found in Table S13.

**Table 1:** Demographic characteristics of participants

	Control	A+/T−	A+/T+
***Participants with concurrent Aβ PET, tau PET, and MRI at baseline (n = 487)***			
A/T/N, *n* (%)	162 (33.3)	143 (29.4)	182 (37.4)
Diagnosis, CU:CI	162:0	93:50	48:134
Females, *n* (%)	102 (63.0)	66 (46.1)^a^	98 (53.8)
Age, years, median (IQR, range)	69.1 (7.7, 55.3–85.0)	74.8 (11.2, 56.6–92.2)^a^	75.7 (10.9, 55.7–94.0)^b^
Age < 65, *n* (%)	21 (13.0)	13 (9.1)	16 (8.8)
Age < 72.7, *n* (%)	116 (71.6)	60 (42.0)^a^	67 (36.8)^b^
Education, years, median (IQR, range)	17 (2, 11–20)	17 (3, 12–20)	16 (4, 12–20)^b,c^
APOE-ε4, *n* (%)	39 (24.7)	65 (47.8)^a^	112 (65.9)^b,c^
APOE-ε2, *n* (%)	19 (12.0)	5 (3.7)^a^	4 (2.4)^b^
KL-VS^het+^, *n* (%)	43 (30.5)	35 (29.9)	24 (19.4)
Aβ PET Centiloids, median (IQR, range)	7.1 (11.2, −17.3–22.4)	47.2 (46.2, 20.7–152.3)^a^	87.0 (55.8, 16.4–253.4)^b,c^
***Participants with ≥2 Aβ PET scans (n = 199)***			
A/T/N, *n* (%)	80 (40.2)	58 (29.1)	61 (30.7)
Diagnosis, CU:CI	80:0	42:16	27:34
Females, *n* (%)	53 (66.3)	24 (41.4)^a^	38 (62.3)
Age, years, median (IQR, range)	69.2 (8.7, 58.4–83.7)	75.5 (8.9, 62.2–91.5)^a^	76.6 (10.7, 56.3–90.4)^b^
Age < 65, *n* (%)	8 (10.0)	3 (5.2)	5 (8.2)
Age < 72.7, *n* (%)	54 (67.5)	20 (34.5)^a^	21 (34.4)^b^
Education, years, median (IQR, range)	17.5 (2, 11–20)	17.0 (4, 12–20)	16.0 (4, 12–20)^b,c^
APOE-ε4, *n* (%)	24 (30.4)	31 (53.4)^a^	36 (59.0)^b^
APOE-ε2, *n* (%)	9 (11.4)	1 (1.7)	3 (4.9)
KL-VS^het+^, *n* (%)	21 (28.4)	13 (23.2)	14 (23.7)
FU visits, median (IQR, range)	2 (0, 2–3)	2 (0, 2–3)	2 (0, 2–)
Duration of FU, years, median (IQR, range)	2.1 (0.4, 1.3–4.2)	2.0 (0.2, 0.8–3.8)	2.1 (0.2, 1.0–3.7)
***Participants with ≥2 tau PET scans (n = 192)***			
A/T/N, *n* (%)	37 (19.3)	71 (37.0)	84 (43.8)
Diagnosis, CU:CI	37:0	46:25	27:57
Females, *n* (%)	23 (62.2)	27 (38.0)^a^	52 (61.9)^c^
Age, years, median (IQR, range)	69.2 (9.3, 62.8–82.7)	73.9 (9.7, 58.2–92.2)^a^	75.8 (10.0, 55.7–89.6)^b^
Age < 65, *n* (%)	6 (16.2)	5 (7.0)	9 (10.7)
Age < 72.7, *n* (%)	26 (70.3)	30 (42.3)^a^	32 (38.1)^b^
Education, years, median (IQR, range)	16 (3, 12–20)	17 (4, 12–20)	16 (4, 12–20)^c^
APOE-ε4, *n* (%)	12 (32.4)	39 (56.5)^a^	56 (70.0)^b^
APOE-ε2, *n* (%)	4 (10.8)	2 (2.9)	2 (2.5)
KL-VS^het+^, *n* (%)	14 (41.2)	19 (31.1)	14 (20.9)
FU visits, median (IQR, range)	2 (1, 2–5)	2 (1, –4)	2 (1, 2–4)
Duration of FU, years, median (IQR, range)	2.0 (1.1, 0.9–3.7)	1.5 (1.0, 0.8–3.6)	1.5 (1.0, 0.8–4.0)

*A/T/N* amyloid-β/tau/neurodegeneration, *CU* cognitive unimpaired, *CI* cognitive impairment, *IQR* interquartile range, *FU* follow-up

^a^Significant difference between the control and A+/T− at *p* < 0.05 with Benjamini-Hochberg corrected

^b^Significant difference between the control and A+/T+ at *p* < 0.05 with Benjamini-Hochberg corrected

^c^Significant difference between A+/T− and A+/T+ at *p* < 0.05 with Benjamini-Hochberg corrected

### Early tau aggregation regions in amyloid-positive individuals

The comparisons of baseline and slopes of Aβ Centiloids and temporal meta-ROI FTP SUVR can be found in Supplementary material (Table S1 and Fig. S7). No significant differences were found in temporal meta-ROI FTP SUVR between the A+/T− and control groups. We subsequently compared the baseline and slopes of FTP SUVRs in each ROI of the temporal meta-ROI among the control, A+/T−, and A+/T+ groups: A+/T+ individuals had significantly higher FTP SUVRs in the amygdala, entorhinal, parahippocampal, fusiform, inferior temporal, and middle temporal cortices than the control group, whereas A+/T− individuals showed significantly or marginally higher FTP SUVRs in the amygdala (*p* < 0.001), entorhinal (*p* = 0.008), and parahippocampal (*p* = 0.059) but not in the fusiform, inferior temporal, and middle temporal cortices when compared to the control group (Fig. S8 and Table S1). Longitudinally, A+/T+ individuals showed significantly faster rates of FTP SUVR increases in entorhinal, parahippocampal, fusiform, inferior temporal, and middle temporal regions than the control and A+/T− groups. Additionally, both A+/T− and A+/T+ individuals had significantly faster rates of FTP SUVR increases in the amygdala than the control group, but no significant difference was found between A+/T− and A+/T+ groups. When we used the alternative cut-off of temporal meta-ROI SUVR (1.27) to define tau positive, the outcomes were substantially the same (Fig. S13 and Table S14).

### Partial correlation of baseline and longitudinal FTP SUVRs in individual ROIs of the temporal meta-ROI

**Fig. 1:**
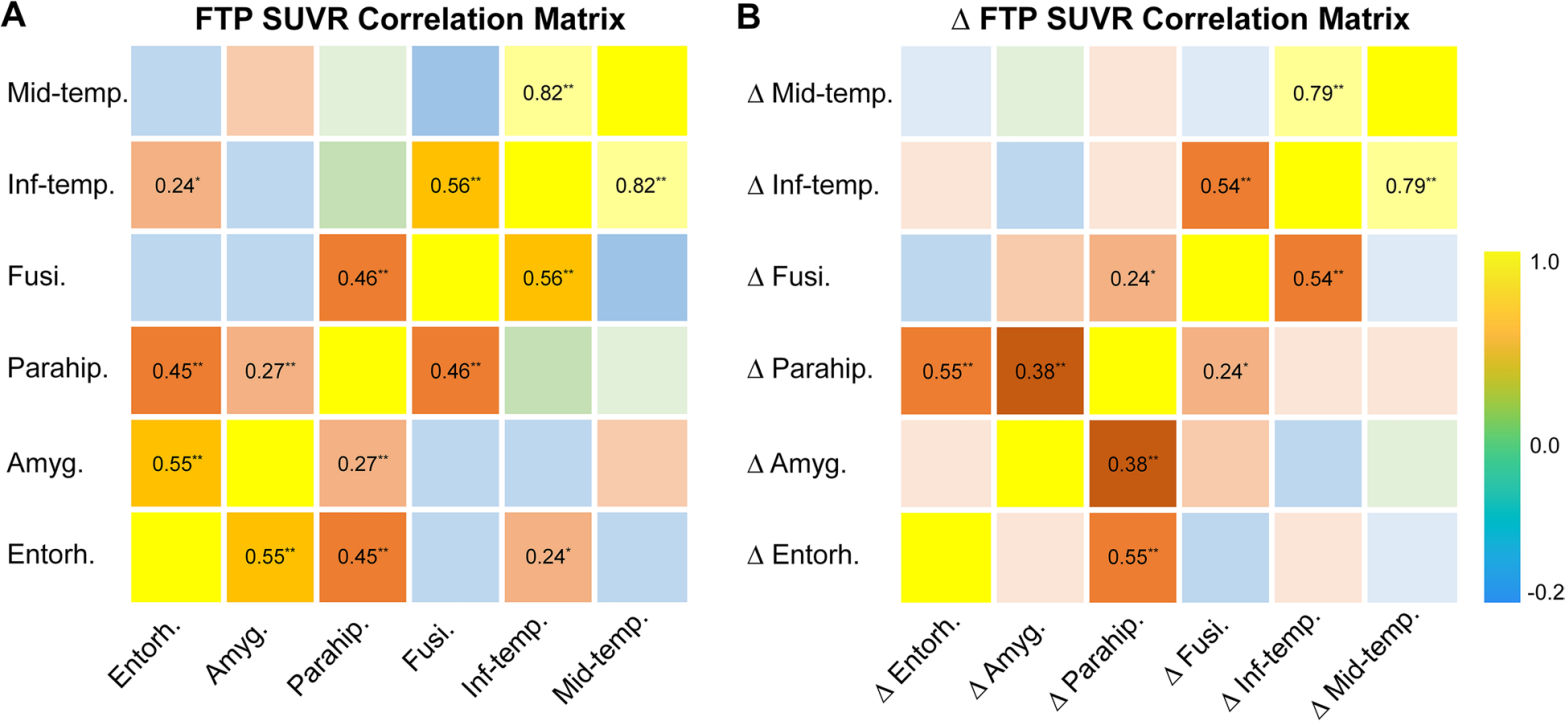
Partial correlation matrices of baseline and annual rates of FTP SUVRs in 6 ROIs within the temporal meta-ROI. Correlation matrices of **A** baseline FTP SUVRs and **B** longitudinal FTP SUVR changes of 6 ROIs within the temporal meta-ROI. Bonferroni corrected for multiple comparisons at *p* < 0.05. Pearson partial correlation coefficients > 0 and survived in Bonferroni correction at *p* < 0.05 and *p* < 0.001 were displayed and marked with single or two asterisks, respectively. Abbreviations: Entoh. entorhinal, Amyg. amygdala, Parahip. parahippocampal, Fusi. fusiform, Inf-temp. inferior temporal, Mid-temp. middle temporal

The regional tau deposition and longitudinal tau accumulation in parahippocampal, fusiform, inferior temporal, and middle temporal regions hierarchically correlated with one another, with the tau tangles of individual ROIs being significantly associated with the tau tangles in adjacent ROIs in the matrices of baseline and slopes of FTP SUVRs. However, the tau tangles in the amygdala and entorhinal did not appear to hold such patterns (Fig. 1 and Tables S2-S3).

### Prediction of longitudinal tau accumulation by baseline Aβ plaques and tau tangles

**Fig. 2:**
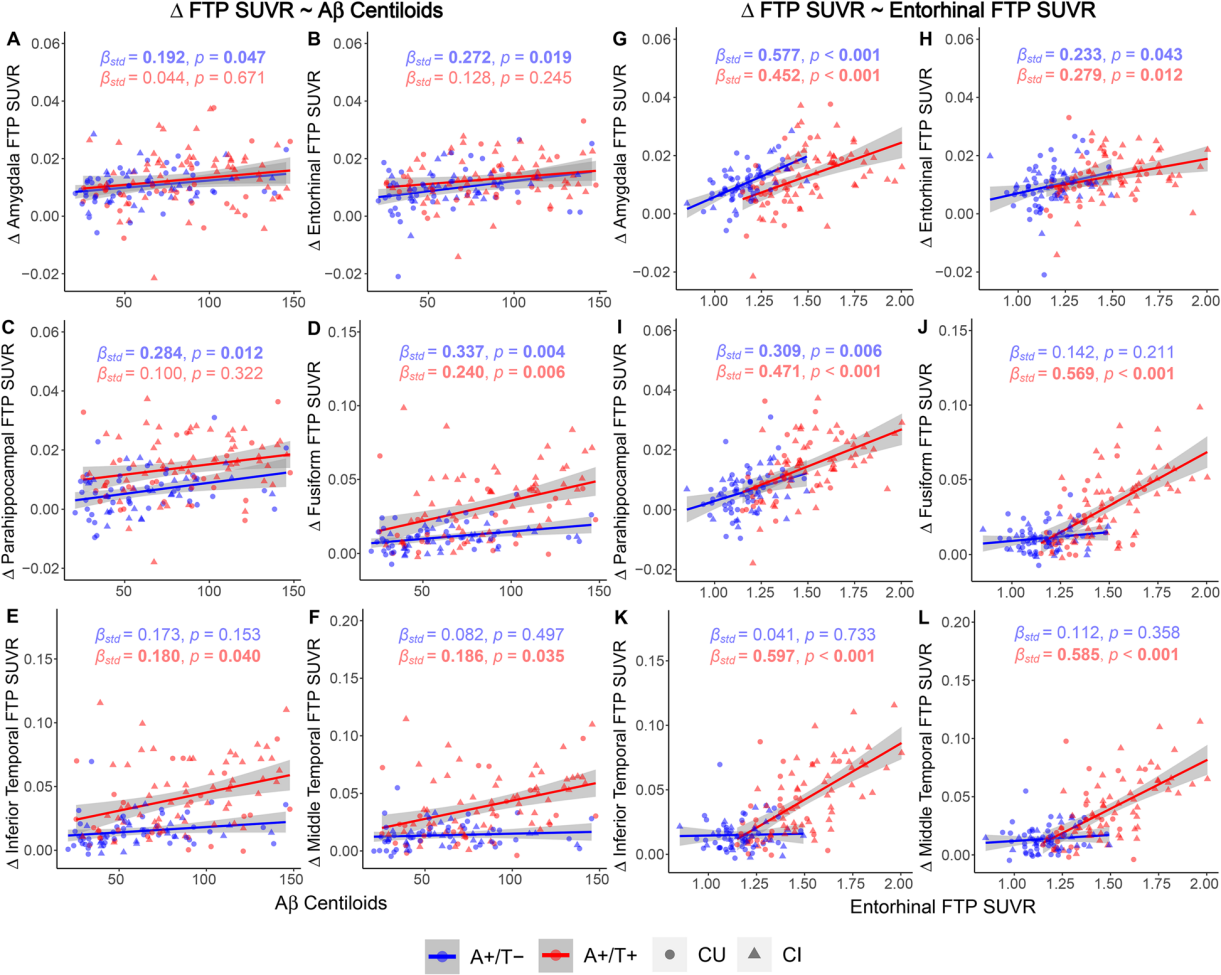
Prediction of longitudinal tau accumulations by baseline Aβ Centiloids and entorhinal tau. Prediction of longitudinal FTP SUVR increases in the amygdala, entorhinal, parahippocampal, fusiform, inferior temporal, and middle temporal regions by baseline **A**–**F** Aβ Centiloids and **F**–**L** entorhinal FTP SUVR. Linear regression lines, datapoints, *β*_std_, and *p* values of A+/T− and A+/T+ groups were colored in blue and red. Circles and triangles, respectively, represented CU and CI individuals. Linear model fits were indicated with 95% confidence intervals. Significant *p* values at *p* < 0.05 and associated *β*_std_ were marked in bold

In A+/T− individuals, higher baseline Aβ Centiloids were significantly associated with faster longitudinal tau increases in the amygdala, entorhinal, parahippocampal, and fusiform cortices but not in inferior temporal or middle temporal cortices (Fig. 2A–F and Tables S4-S6) regardless of which regional FTP SUVR was selected as the simultaneous predictor. However, in A+/T+ individuals, baseline Aβ Centiloids were predictive of longitudinal tau increases in fusiform, inferior temporal, and middle temporal cortices but not in amygdala, entorhinal, or parahippocampal cortices after accounting for amygdala or entorhinal FTP SUVR. Furthermore, when including the FTP SUVR of parahippocampal, fusiform, inferior temporal, or middle temporal cortices as variables, we did not see any meaningful predictive effects of baseline Aβ Centiloids on longitudinal tau increases (Tables S5-S6).

**Fig. 3:**
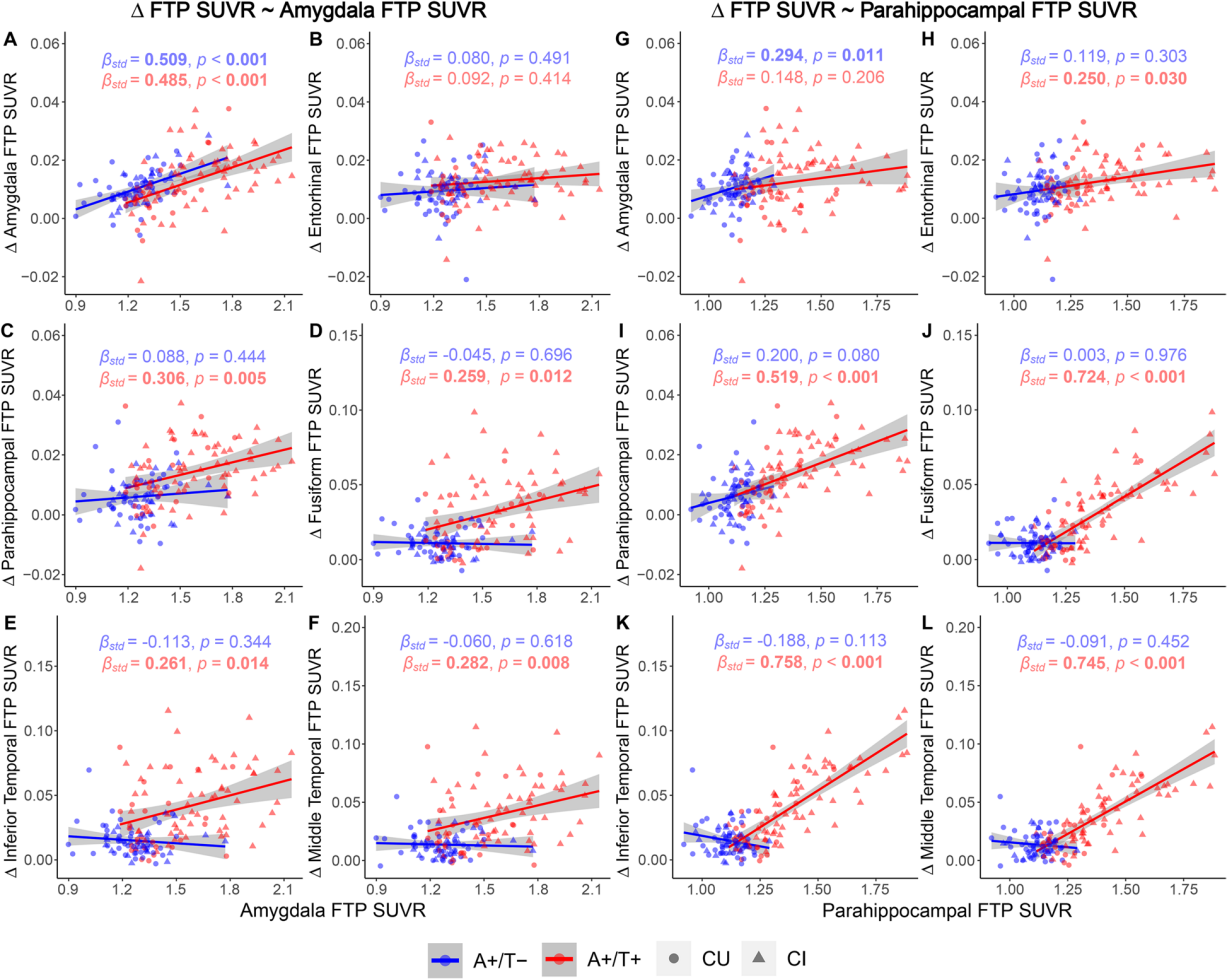
Prediction of longitudinal tau accumulations by baseline tau in the amygdala and parahippocampal gyrus. Prediction of longitudinal FTP SUVR increases in the amygdala, entorhinal, parahippocampal, fusiform, inferior temporal, and middle temporal regions by baseline FTP SUVR in **A**–**F** amygdala and **G**–**L** parahippocampal gyrus. Linear regression lines, datapoints, *β*_std_, and *p* values of A+/T− and A+/T+ groups were colored in blue and red. Circles and triangles, respectively, represented CU and CI individuals. Linear model fits were indicated with 95% confidence intervals. *p* < 0.05 and associated *β*_std_ were marked in bold

Although higher entorhinal FTP SUVR only predicted faster rates of FTP SUVR increases in the amygdala, entorhinal, and parahippocampal cortices in A+/T− individuals, it did demonstrate predictive effects in longitudinal tau increases in all ROIs within temporal meta-ROI in A+/T+ individuals (Fig. 2G–L and Table S4). Only in the amygdala did higher baseline amygdala FTP SUVR predict faster FTP SUVR increases in A+/T− individuals, but for A+/T+ individuals, higher baseline amygdala FTP SUVR was predictive of more rapid longitudinal tau accumulation in all ROIs except for entorhinal cortex (Fig. 3A–F and Table S4). In A+/T− individuals, higher baseline parahippocampal FTP SUVR indicated faster longitudinal tau increase exclusively in the amygdala, whereas in A+/T+ individuals, longitudinal rates of tau accumulation in all ROIs except for the amygdala could be predicted (Fig. 3G–L and Table S5). Additionally, baseline FTP SUVRs in fusiform, inferior temporal, and middle temporal cortices predicted faster tau accumulations in parahippocampal, fusiform, inferior temporal, and middle temporal cortices in A+/T+ individuals but not in A+/T− individuals (Tables S5-S6 and Fig. S9).

Results were substantially the same as we investigated the association between baseline Aβ Centiloids and regional rates of tau accumulation in the whole cohort and in T− and T+ subgroups (results not shown). We also found similar results as we applied the alternative temporal meta-ROI FTP SUVR (1.27) threshold (Fig. S14-S15 and Tables S15-S17).

### Association of sex, age, baseline Aβ plaques, and tau tangles with longitudinal tau accumulation

No significant sex effect was found in Aβ-related annual FTP SUVR changes. Interestingly, we detected that the faster longitudinal fusiform FTP SUVR increase in females than in males could be significantly predicted by baseline FTP SUVR in the middle temporal region (*p* = 0.008) and marginally indicated by baseline FTP SUVRs in the inferior temporal (*p* = 0.054) and fusiform (*p* = 0.100) areas (Table S7 and Fig. S10).

**Fig. 4:**
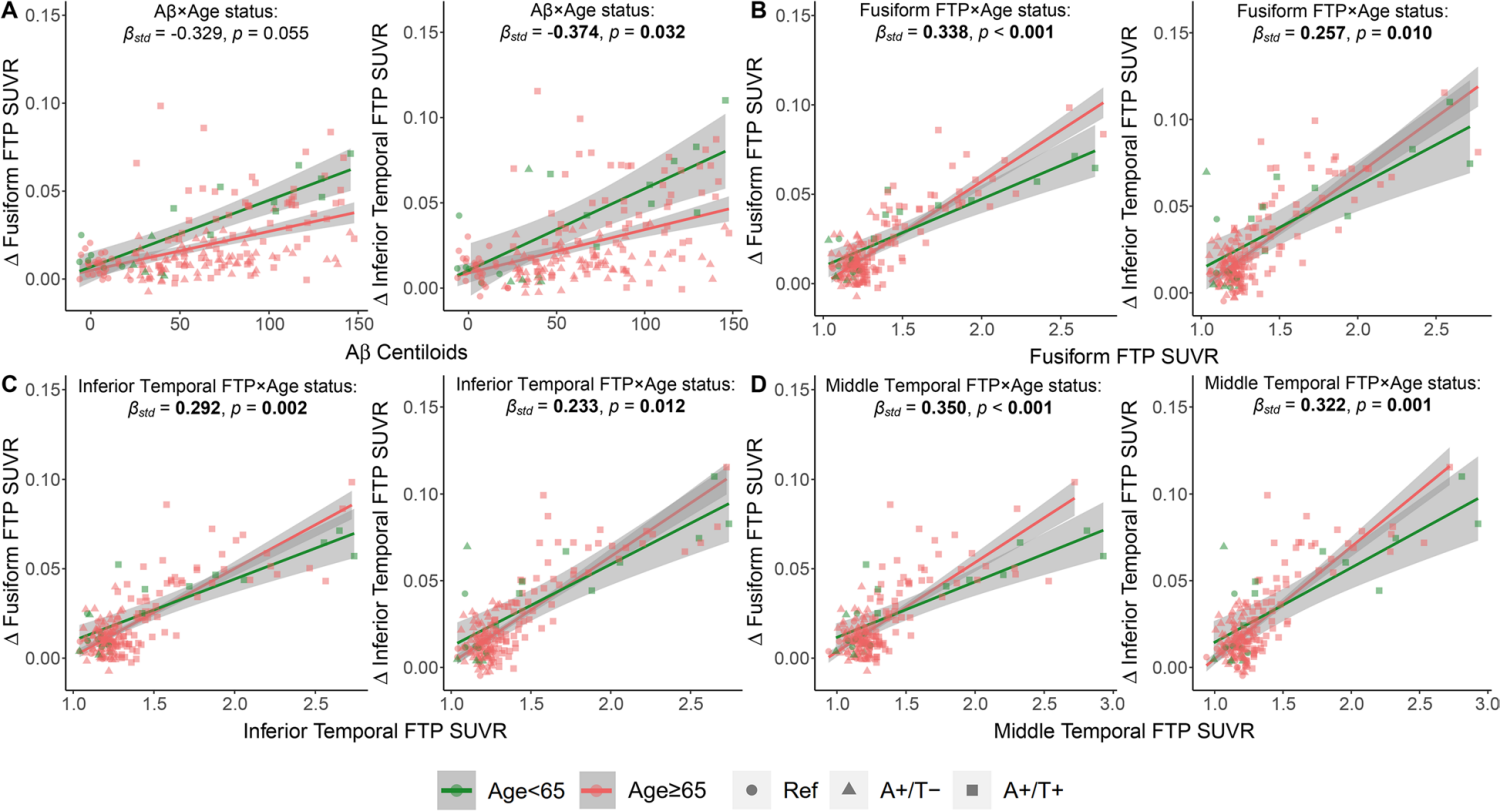
Aβ- and tau-related longitudinal tau accumulation in early-life and late-life elderly adults. Significant negative interaction of **A** age<65 × Aβ, and positive interactions of **B** age<65 × fusiform FTP SUVR, **C** age<65 × inferior temporal FTP SUVR, **D** age<65 × middle temporal FTP SUVR on longitudinal tau increases in fusiform and inferior temporal region. Linear regression lines and datapoints of age < 65 and age ≥ 65 adults were colored in salmon and green, and the control, A+/T−, and A+/T+ participants were represented by circles, triangles, and squares, respectively. Linear model fits were indicated with 95% confidence intervals. *p* < 0.05 and associated *β*_std_ were marked in bold

Importantly, we found that late-life elderly adults (age ≥ 65) exhibited slower Aβ-related longitudinal tau increases in fusiform (*p* = 0.055), inferior temporal (*p* = 0.032), and middle temporal areas (*p* = 0.013) than early-life elderly adults (age < 65) (Fig. 4A, Table S8, and Fig. S11A). In contrast, higher tau levels in fusiform, inferior temporal, and middle temporal regions could predict higher rates of tau accumulation in fusiform and inferior temporal (Fig. 4B–D) as well as parahippocampal cortices (Fig. S11C-E) in early-life elderly adults than in late-life elderly adults. Besides, compared to early-life elderly adults, late-life elderly adults also showed faster parahippocampal tau-associated longitudinal tau increase in the parahippocampal gyrus (Fig. S11B), fusiform tau-associated longitudinal tau increase in the amygdala (Table S8), and middle temporal tau-associated longitudinal tau increase in the middle temporal region (Fig. S11F).

Results substantially remained the same as when we used the alternative cut-off age of 72.7 years (Table S9 and Fig. S11G-L).

### Association of KL-VS^het^, APOE-ε4, baseline Aβ plaques, and tau tangles with longitudinal tau accumulation

**Fig. 5:**
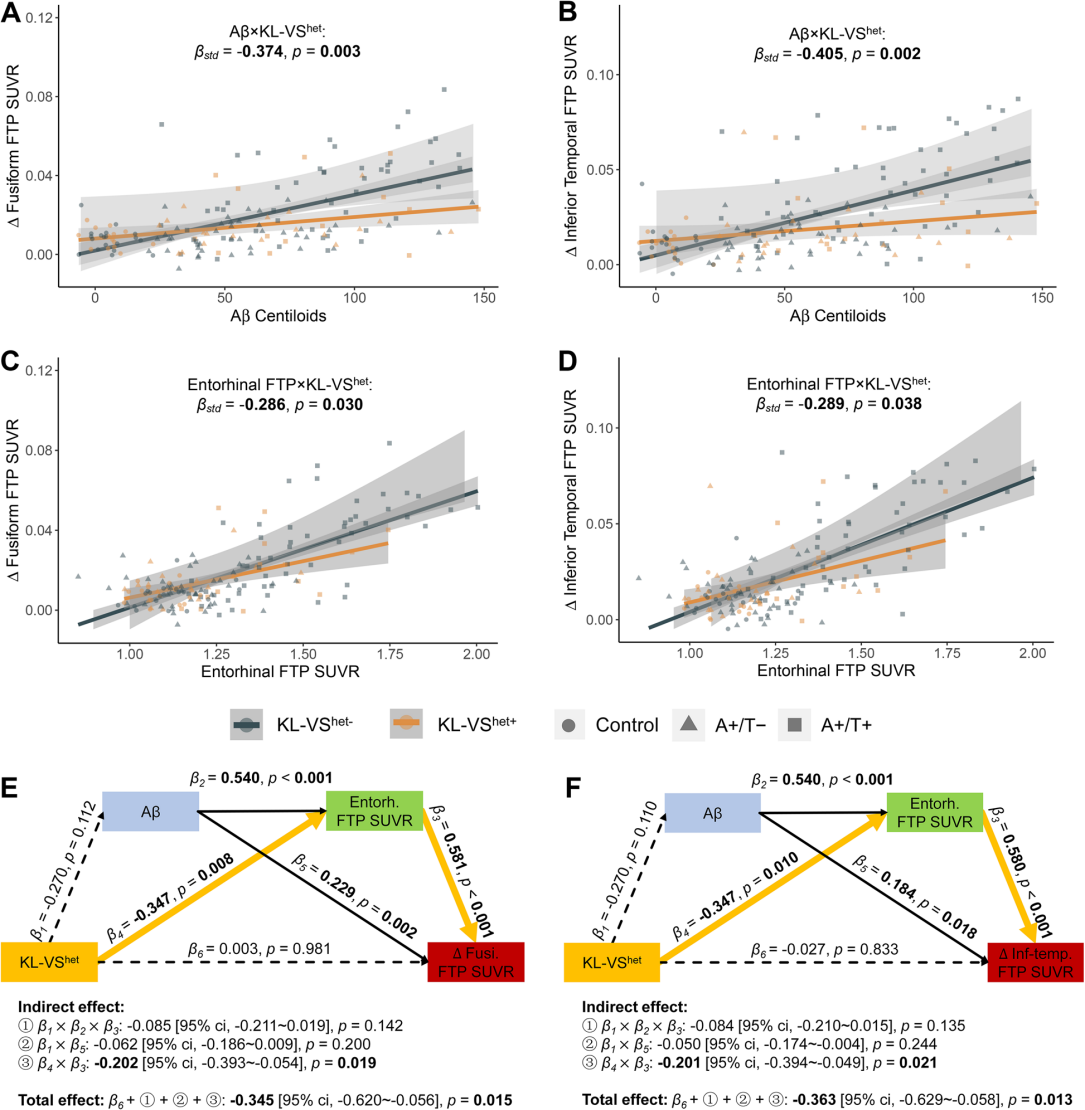
The influences of KL-VS^het^ genotype (KL-VS^het−^ vs. KL-VS^het+^) on longitudinal tau propagations. Negative interaction of KL-VS^het−^ × Aβ in longitudinal tau propagation to **A** fusiform and **B** inferior temporal region. Negative interaction of KL-VS^het−^ × entorhinal FTP SUVR in longitudinal tau propagation to **C** fusiform and **D** inferior temporal region. Possible pathways among KL-VS^het^, baseline Aβ Centolids, entorhinal tau concentration, and longitudinal tau accumulations in **E** fusiform and **F** inferior temporal region. ① *β*_1_ × *β*_2_ × *β*_3_, ② *β*_1_ × *β*_5_, and ③ *β*_4_ × *β*_3_ represented three indirect pathways, and *β*_6_ + ① + ② + ③ represented the total effect. All variables have been converted to standard *z*-scores. Total, direct, and indirect associations were calculated based upon a 5000-iteration bootstrapping procedure. Linear regression lines and datapoints of KL-VS^het−^ and KL-VS^het+^ participants were colored in gray and orange, and the control, A+/T−, and A+/T+ participants were represented by circles, triangles, and squares, respectively. Linear model fits were indicated with 95% confidence intervals. Significant *p* values at *p* < 0.05 and associated *β*_std_ were marked in bold. Solid and dashed lines showed significant and insignificant associations, and the significant indirect pathways were characterized by bold orange lines

In comparison with KL-VS^het−^ individuals, KL-VS^het+^ individuals showed lower baseline FTP SUVRs in temporal meta-ROI and all individual ROIs included and slower rates of FTP SUVRs in temporal meta-ROI and all individual ROIs except for amygdala (Table S10 and Fig. S12B-H). On the other hand, there was no discernible difference between KL-VS^het+^ and KL-VS^het−^ individuals regarding the baseline or slope of Aβ Centiloids (Table S10 and Fig. S12A). The KL-VS^het+^/KL-VS^het−^ ratio was found to be 0.820 in temporal meta-ROI, 0.892 in amygdala, 0.755 in entorhinal, 0.551 in parahippocampal, 0.856 in fusiform, 0.757 in inferior temporal, and 0.679 in middle temporal cortices (Table S11). In other words, KL-VS^het+^ individuals had 55.1–82.0% times lower rates of tau accumulation than KL-VS^het−^ individuals. Furthermore, KL-VS^het+^ individuals had attenuated Aβ-related longitudinal tau accumulations in parahippocampal (*p* = 0.016), fusiform (*p* = 0.003), inferior temporal (*p* = 0.002), and middle temporal (*p* = 0.007) regions than KL-VS^het−^ individuals (Fig. 5A, B and Table S12). More importantly, slower entorhinal tau-associated longitudinal tau increases were found in fusiform (*p* = 0.030) and inferior temporal lobe (*p* = 0.038) in KL-VS^het+^ individuals than in KL-VS^het−^ individuals (Fig. 5C, D and Table S12).

Since we found that both Aβ-related and entorhinal tau-related longitudinal tau accumulations in fusiform and inferior temporal regions were significantly attenuated in KL-VS^het+^ individuals, we conducted a further mediation analysis to investigate the association among KL-VS^het^, Aβ Centiloids, entorhinal FTP SUVR, and longitudinal changes of FTP SUVRs in fusiform and inferior temporal regions. From the analysis, we observed that the direct association between KL-VS^het^ and ∆ FTP SUVRs in fusiform and inferior temporal area became insignificant after incorporating baseline Aβ Centolids and entorhinal FTP SUVR into the model. Also, the negative *β*_std_ values changed from −0.345 to 0.003 (101% change) and from −0.363 to −0.027 (92.6% change), respectively (Fig. 5E, F). Thus, it is suggested that the attenuated longitudinal tau accumulations in fusiform and inferior temporal regions were significantly mediated by baseline entorhinal FTP SUVR.

In contrast, APOE-ε4 was not found to affect the prediction of longitudinal tau accumulations in temporal meta-ROI and all individual ROIs within the composite region using baseline Aβ plaques or tau tangles.

## Discussion

In this study, we investigated how cortical tau tangles propagate in MTL, inferior, and middle temporal cortices in AD. We found that initial levels of Aβ and tau tangles were linked differently to the longitudinal tau accumulation inside and outside of MTL. Specifically, baseline Aβ plaques and entorhinal tau tangles were related to faster tau accumulation in the amygdala, entorhinal, and parahippocampal gyrus included in the temporal meta-ROI in A+/T− individuals. While greater initial entorhinal tau deposition predicted faster tau accumulations in MTL and further propagations into inferior and middle temporal cortices, higher baseline Aβ plaques were only poorly associated with faster FTP SUVR increases in fusiform, inferior temporal, and middle temporal regions in A+/T+ individuals. Furthermore, we found that early-life elderly adults had faster Aβ-dependent but slower tau-dependent longitudinal tau accumulations in fusiform and inferior temporal areas than late-life elderly adults. Additionally, we found that KL-VS^het+^ attenuated longitudinal tau accumulations in fusiform and inferior temporal lobe via modulating initial entorhinal tau-associated rather than Aβ-associated tau propagation. These findings are crucial for understanding how tau tangles propagate in the early Braak stages of AD, providing novel insights to design future AD clinical trials.

We first determined the early tau-deposited regions in individuals with abnormal AD summary cortical Aβ PET SUVR but normal temporal meta-ROI tau PET SUVR (A+/T−). Focusing on this group, we found that A+/T− individuals had significantly or marginally higher baseline FTP SUVRs and faster rates of FTP SUVR increases in the amygdala, entorhinal, and parahippocampal gyrus compared to A−/T−/N− individuals. The partial correlation analysis further revealed a potential cascading order of tau spread from parahippocampal gyrus to fusiform to inferior temporal to middle temporal cortices. In contrast, the propagating sequence was unambiguous in the entorhinal and amygdala. Early tau deposition likely occurs in the entorhinal and amygdala in the early stages of AD. Consistently, one recent study also observed the most considerable tau deposited in the amygdala in both A−/T+ and A+/T+ individuals [38], and numerous studies [11, 13, 39–41] have reported the entorhinal cortex being the earliest site of neurofibrillary tau tangle formation.

The Harvard group [11] recently observed that higher baseline entorhinal tau and Aβ burden would trigger the greatest tau spreading into the inferior temporal region. Another recent study also highlighted the significant interaction of Aβ plaques and tau tangles in promoting the onset and acceleration of longitudinal tau accumulation [23]. Congruently, our findings showed that for A+/T− individuals, both baseline Aβ plaques and entorhinal tau tangles were associated with faster tau accumulation in early tau-deposited ROIs (amygdala, entorhinal, and parahippocampal) of temporal meta-ROI. These findings provide further evidence to explain how initial Aβ burden and regional tau deposition are associated with longitudinal tau accumulation in MTL in Aβ-positive individuals without abnormal temporal meta-ROI tau. Unlike the initial entorhinal tau level, initial amygdala and parahippocampal tau levels in A+/T− individuals only predicted faster tau accumulation in amygdala, and the tau levels in late tau-deposited ROIs (fusiform, inferior temporal, and middle temporal cortices) of temporal meta-ROI did not predict any longitudinal tau accumulation in early tau-deposited regions. These findings suggest that the faster tau accumulations in amygdala, entorhinal, and parahippocampal gyrus are probably associated with existing cortical Aβ plaques and entorhinal tau deposition in the A+/T− stage of AD.

Once individuals had widespread tau tangles in temporal meta-ROI (A+/T+), unlike tau levels in other individual ROIs in temporal meta-ROI, higher initial entorhinal tau level was positively correlated with faster tau accumulations in all individual ROIs. Such findings suggest that entorhinal tau might be a crucial predictor of the subsequent accumulation of tau aggregates in mesial and basal temporal neocortices [11]. Notably, for A+/T+ individuals, the baseline tau levels and longitudinal tau accumulations were strongly linked to each other in the relatively late tau-deposited ROIs (parahippocampal, fusiform, inferior temporal, and middle temporal regions). However, the tau levels in relatively late tau-deposited ROIs would unlikely affect tau accumulations in early tau-deposited regions, whether in A+/T− or A+/T+ groups. The prediction effect of faster tau accumulation in fusiform, inferior temporal, and middle temporal areas by a more considerable Aβ burden became minimal no matter the tau level of which late tau-deposited ROI was included in our models.

Notably, our findings are not suggesting that existing Aβ plaques are unimportant for longitudinal tau accumulation. Recently, Lee and colleagues [23] observed a significant local interaction of Aβ plaques and tau tangles in the inferior temporal region, sparking the most significant acceleration of tauopathy. Differences in modeling methodology might underlie such discrepancies. While the local Aβ and tau interaction (Aβ×tau) within the same region was modeled in their study [23], we used a summary cortical Aβ PET SUVR of several brain areas [32] to represent cortical Aβ burden. The amount of Aβ plaques and regional tau tangles were modeled separately to distinguish their respective contributions in the downstream tau accumulation. Furthermore, many studies reported that abnormal tau tangles outside the entorhinal cortex were rarely observed in the absence of substantial cortical Aβ burden [9, 20, 21, 25, 42], and larger Aβ burden was strongly related to more rapid tau accumulation over time [5, 10, 11, 20]. Altogether, it might be possible that the existing tau tangles contribute more to longitudinal tau accumulations in late tau-deposited ROIs of temporal meta-ROI than cortical Aβ plaques when Aβ plaques and tau tangles are already widespread in neocortex and temporal meta-ROI.

Our findings may have potential implications for AD clinical trials (e.g., anti-Aβ or anti-tau treatments): For Aβ+ individuals without abnormal temporal meta-ROI tau, reducing cortical Aβ plaques might be a promising therapeutic strategy to decelerate the further spreading of tau aggregates, and prevent subsequent tau-associated neurodegeneration and cognitive decline. However, for individuals who already have widespread cortical Aβ plaques and tau tangles, sole Aβ-lowering therapy might be insufficient. Directly targeting the local tau aggregates might be more effective in preventing AD progression.

Recent literature has demonstrated that females have more tau depositions [30] and faster tau accumulations [24, 29] than males. One cross-sectional study reported that the tau tangles of females in the entorhinal and inferior temporal lobe were more positively associated with Aβ burden than those of males [43]. Similarly, our work further manifested that females may exhibit faster initial tau-associated rather than Aβ-associated longitudinal tau increases than males. Both cross-sectional [44] and longitudinal studies [24, 26, 29] have found that larger amounts of Aβ plaques were associated with faster tau accumulation in Aβ+ individuals under 65 years old. Congruently, we also observed more rapid Aβ-associated longitudinal tau increases in the fusiform, inferior temporal, and middle temporal cortices in early-life elderly adults (age < 65 or age < 72.7 years) than in late-life elderly adults (age ≥ 65 or age ≥ 72.7 years). Unexpectedly, participants of older ages showed faster tau-related longitudinal tau increases in parahippocampal, fusiform, inferior temporal, and middle temporal cortices compared to those of younger ages. To the best of our knowledge, this primary finding has not been thoroughly discussed in the literature. In line with our result, a recent animal study found that older animals had more tau spreading in the hippocampus and adjacent cortices than younger ones [45].

Previous findings have found that KL-VS^het+^ carriers have decreased Aβ and tau pathologies [46]. For instance, in one recent study, KL-VS^het+^ carriers attenuated Aβ-associated tau increases cross-sectionally and longitudinally [28]. Similarly, for KL-VS^het+^ carriers, our results exhibited marginally decreased Aβ levels but prominently decreased baseline tau levels and rates of tau accumulation (e.g., KL-VS^het+^/KL-VS^het−^ ratio as 0.551 in the parahippocampal gyrus). This reduction effect might indicate a notable lowering effect of KL-VS^het+^ genotyping on subsequent tau accumulations. More specifically, in our current study, we observed both Aβ-associated and entorhinal tau-associated decreased longitudinal tau accumulation in fusiform and inferior temporal cortices in KL-VS^het+^ carriers, suggesting that KL-VS^het+^ carriers might slow down both Aβ-associated and tau-associated longitudinal tau accumulation in AD. Furthermore, mediation analyses verified that only the pathway from KL-VS^het^ to initial entorhinal tau, then to longitudinal tau accumulation, was statistically significant when we included baseline levels of Aβ plaques and tau aggregates into the models. This verified pathway demonstrates that the slower rate of tau accumulation in KL-VS^het+^ carriers was probably mediated via decreased entorhinal tau-associated rather than Aβ-associated pathway. The findings reached by the current study extended our knowledge regarding the potential protective mechanism of KL-VS^het+^ genotyping and might have implications for the selection of at-risk individuals for clinical trials.

Notably, the influences of sex, age, and KL-VS^het^ on longitudinal tau accumulation are predominantly tau-dependent, and the brain regions involved are mainly located in the late tau-deposited ROIs of temporal meta-ROI. Our preceding findings demonstrated that both Aβ burden and tau tangles act in the early stage of tauopathy, while subsequent accumulation of tau aggregates may strongly associate with baseline levels of tau aggregates at a more advanced stage of AD. Therefore, we infer that sex, age, and KL-VS^het^ mainly modulate the relationship between Aβ burden, tau tangles, and longitudinal tau accumulation at a stage when tau tangles accumulate rapidly and when initial symptoms of AD start to emerge [23, 47, 48].

Several limitations should be addressed for our current work. As we know, temporal meta-ROI has been regarded as an early composite region of tau aggregation formation in AD according to post-mortem [13] and neuroimaging [14, 15] studies, and this composite region, therefore, has been commonly used to detect early tau depositions [5, 24, 49, 50]. Nevertheless, several recent studies highlighted the heterogeneity of tau spreading patterns [22, 51–53]. Thus, it still requires further investigation to confirm whether the findings in the present study can extend to distinct trajectories of tau spreading in AD. Secondly, the interpretations of the findings related to sex, age, and KL-VS^het^ should be cautious as we used only one observational cohort and may need to be confirmed in other independent cohorts. Additionally, such a limited sample size constrained our capability to investigate the effects of baseline Aβ plaques/tau tangles on longitudinal tau accumulation in A+/T− and A+/T+ profiles, which might also be modulated by sex, age, and genotyping factors. Thus, future studies with a larger sample size of longitudinal tau PET would be beneficial.

Overall, our findings suggest that initial levels of Aβ plaques and tau tangles might be differently linked to the further spreading of tau aggregates in the early Braak stage of AD. Specifically, both initial Aβ plaques and tau tangles may be related to faster tau accumulation in early tau-deposited regions (entorhinal, amygdala, and parahippocampal gyrus) for A+/T− individuals, whereas further longitudinal tau propagation in late tau-deposited regions (fusiform, inferior temporal and middle temporal lobes) is mainly associated with the existing tau tangles for A+/T+ individuals. Furthermore, age, sex, and KL-VS^het^ all play a role in modulating the relationships between baseline levels of Aβ plaques/tau tangles and subsequent tau accumulation. These findings provide novel insights into understanding the spatial and temporal patterns of tau accumulation in AD and highlight the effects of biological stages, Aβ plaques, tau tangles, age, sex, and KL-VS^het^ genotyping on longitudinal tau accumulation.

## Supplementary Information

**Additional file 1: Supplemental Methods.** MRI imaging processing. **Supplemental Figures and Tables.** **Figures S1-16, Tables S1-17.** **Supplemental References**.

## Data Availability

The dataset supporting the conclusions of this article is available in the ADNI repository (ida.loni.usc.edu). Derived data is available from the corresponding author on request by any qualified investigator subject to a data use agreement.

## Abbreviations

Aβ: β-Amyloid
AD: Alzheimer’s disease
ADNI: Alzheimer’s Disease Neuroimaging Initiative
Amygd.: Amygdala
AUC: Area under the curve
CI: Cognitive impaired
ci: Confidence interval
CU: Cognitively unimpaired
Entorh: Entorhinal
FBB: ^18^F-florbetaben
FBP: ^18^F-florbetapir
FTP: ^18^F-flortaucipir
FU: Follow-up
Fusi: Fusiform
GLM: Generalized linear models
HCV: Hippocampal volume
ICV: Intracranial volume
Inf-temp: Inferior temporal
IQR: Interquartile range
LME: Linear mixed effect
MCI: Mild cognitive impairment
Mid-temp: Middle temporal
Parahip: Parahippocampal
rHCV: Residual hippocampal volume
ROC: Receiver operating characteristic curve
ROI: Region of interest
SUVR: Standardized uptake value ratio
