# Supplementary material for: Initial levels of β-amyloid and tau deposition have distinct effects on longitudinal tau accumulation in Alzheimer’s disease
Source: Alzheimers Res Ther. 2023 Feb 7;15:30. doi: 10.1186/s13195-023-01178-w (PMC9903587; doi:10.1186/s13195-023-01178-w)
Supplement: Supplementary file 1 — Additional file 1: Supplemental Methods. MRI imaging processing. Supplemental Figures and Tables. Figures S1-16, Tables S1-17. Supplemental References. [file 13195_2023_1178_MOESM1_ESM.pdf]

Initial levels of β-amyloid and tau deposition have distinct effects on longitudinal tau accumulation in Alzheimer's disease

Supplementary material

Supplemental Methods

MRI imaging processing

Supplemental Figures and Tables

Figures S1-16, Tables S1-17

Supplemental References

Supplemental Methods

MRI imaging processing

T1-weighted anatomical MRI images were acquired on Siemens, GE or Philips MRI scanners according to a standard protocol (8), and more details can be found elsewhere (http://adni-info.org). The most fully pre-processed format anatomical MRI images were downloaded from the LONI website (ida.loni.usc.edu). Hippocampal volume (HCV) (cm^3) was calculated across hemispheres from the anatomical MRI scan that was closest to baseline FTP PET scan using FreeSurfer and adjusted by the estimated total intracranial volume (ICV) using the approach reported by the Mayo group (9). The residual HCV (rHCV) was calculated as the difference between the raw HCV and the expected HCV as we described previously (10). Cortical thickness in 68 ROIs were also calculated using FreeSurfer, and the mean temporal meta-ROI cortical thickness was calculated as a surface-area weighted average of the mean cortical thickness in individual ROI of the temporal meta-ROI region. The thresholds of residual hippocampal volume (rHCV) and temporal meta-ROI cortical thickness were set as $\leq$-0.67 cm^3 and $\leq$2.60 mm respectively according to receiver operating characteristic curve (ROC) analysis using the Youden index classifying 435 A- ADNI CU participants, 579 A+ ADNI MCI and AD patients as the endpoint as described in the Supplement (Figure S3-S6).

Supplemental Figures and Tables

Cutoff of temporal meta-ROI FTP SUVR

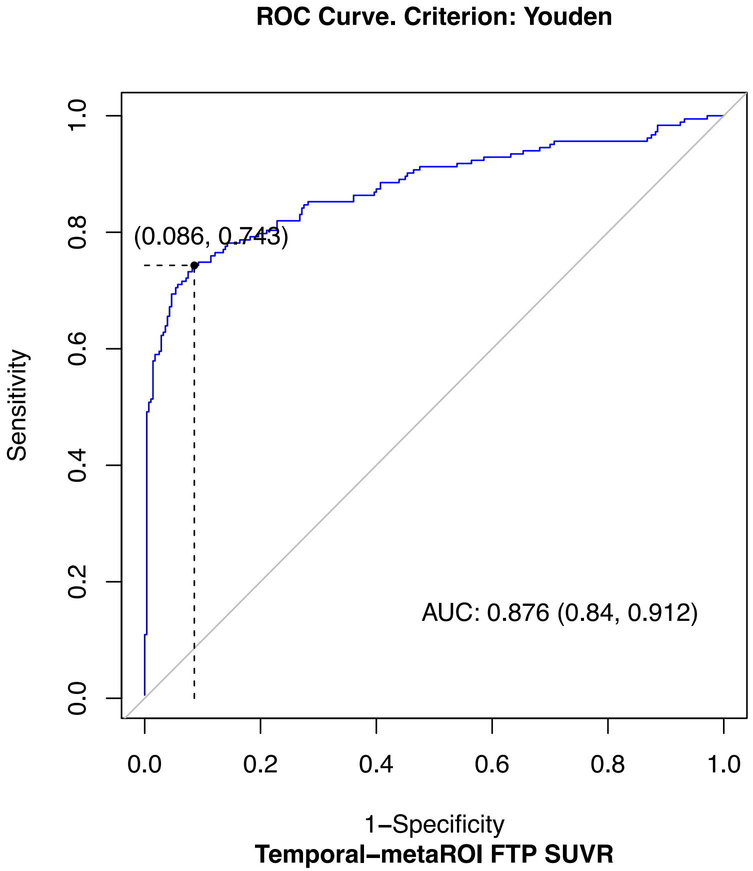

Figure S1. The ROC (receiver operating characteristic curve) analysis using cutoff ≥ 1.25 for temporal meta-ROI FTP SUVR. The analysis using the Youden index classifying 280 Aβ- ADNI cognitively unimpaired (CU) participants and 183 Aβ+ ADNI MCI (mild cognitive impairment) and Alzheimer's disease (AD) patients as the endpoint to define the cutoff ⩾1.25 for temporal meta-ROI FTP SUVR. AUC (area under the curve): 0.876 [95% ci, 0.84 ~ 0.912].

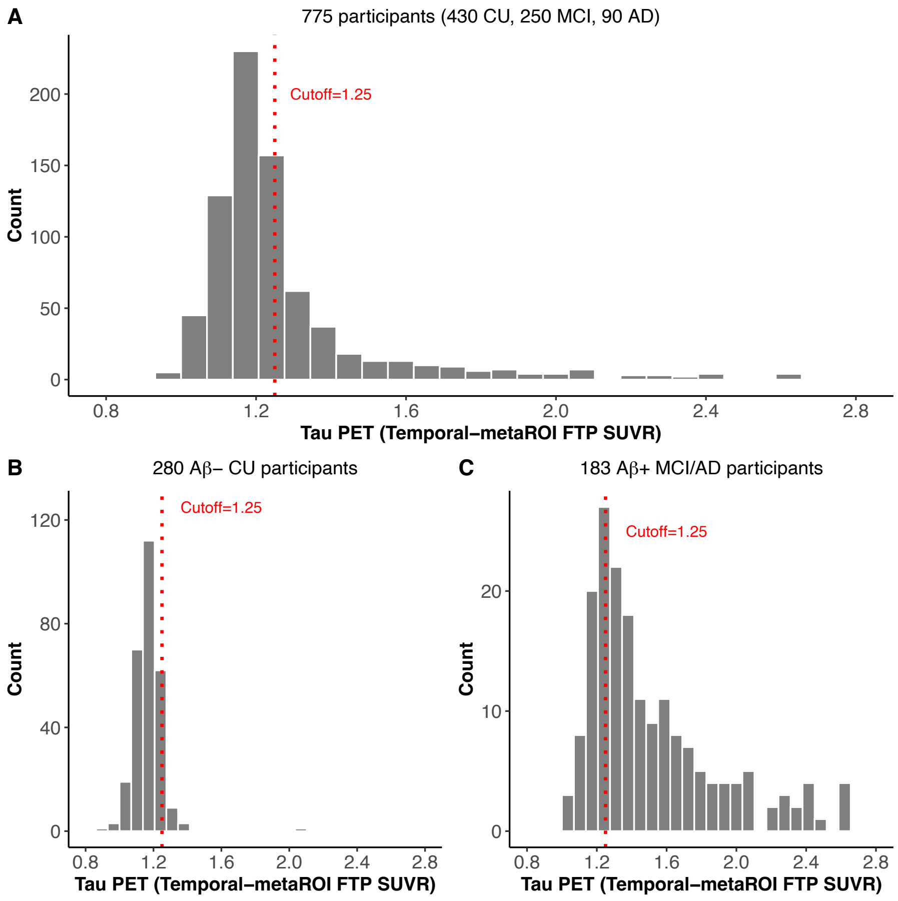

Figure S2. Histograms of temporal meta-ROI FTP SUVRs within different groups. (A) All 775 ADNI participants, **(B)** 280 Aβ- ADNI CU participants and **(C)** 183 Aβ+ ADNI MCI and AD patients with tau PET scan. Red dotted line is the cutoff of temporal meta-ROI FTP SUVR (1.25).

Cutoff of residual hippocampal volume (rHCV)

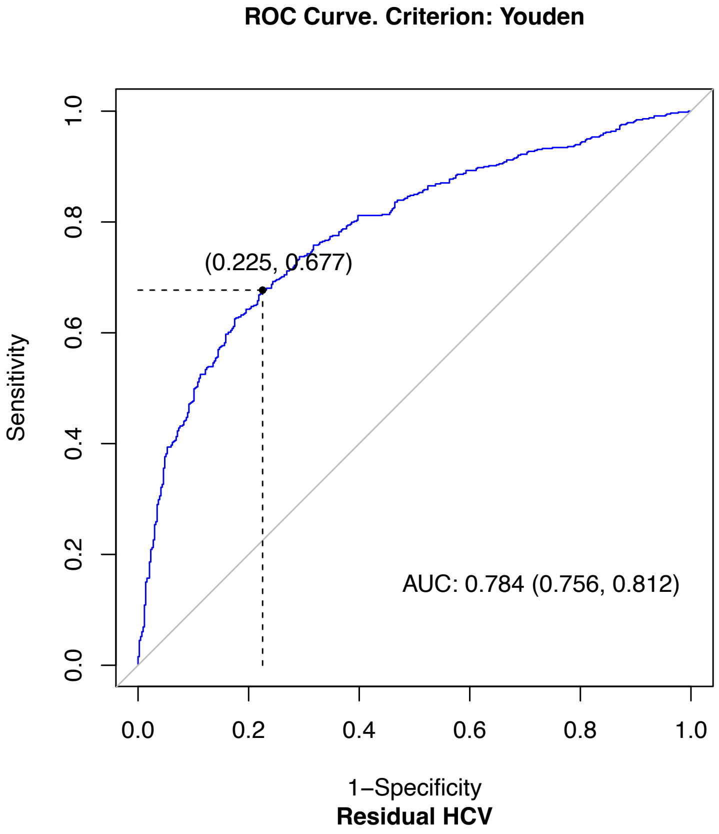

Figure S3. The ROC analysis using cutoff <= -0.67 cm³ for rHCV. The analysis using the Youden index classifying ADNI 435 Aβ- CU participants and 579 Aβ+ MCI and AD patients as the endpoint to define the cutoff <=-0.67 cm³ for rHCV. AUC: 0.78 [95% ci, -0.756 ~ 0.812].

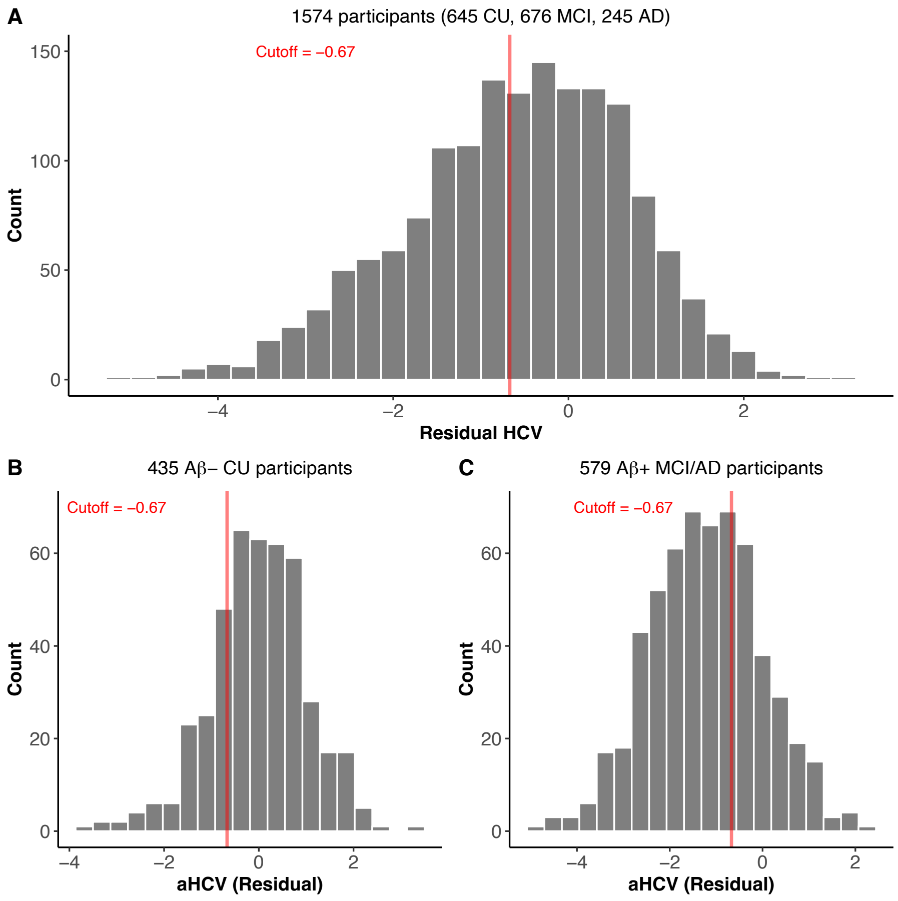

Figure S4. Histograms of rHCVs within different groups. (A) All 1574 ADNI participants, **(B)** 435 Aβ- ADNI CU participants and **(C)** 579 Aβ+ ADNI MCI and Alzheimer's disease patients with rHCV data. Red dotted line is the cutoff of rHCV -0.67.

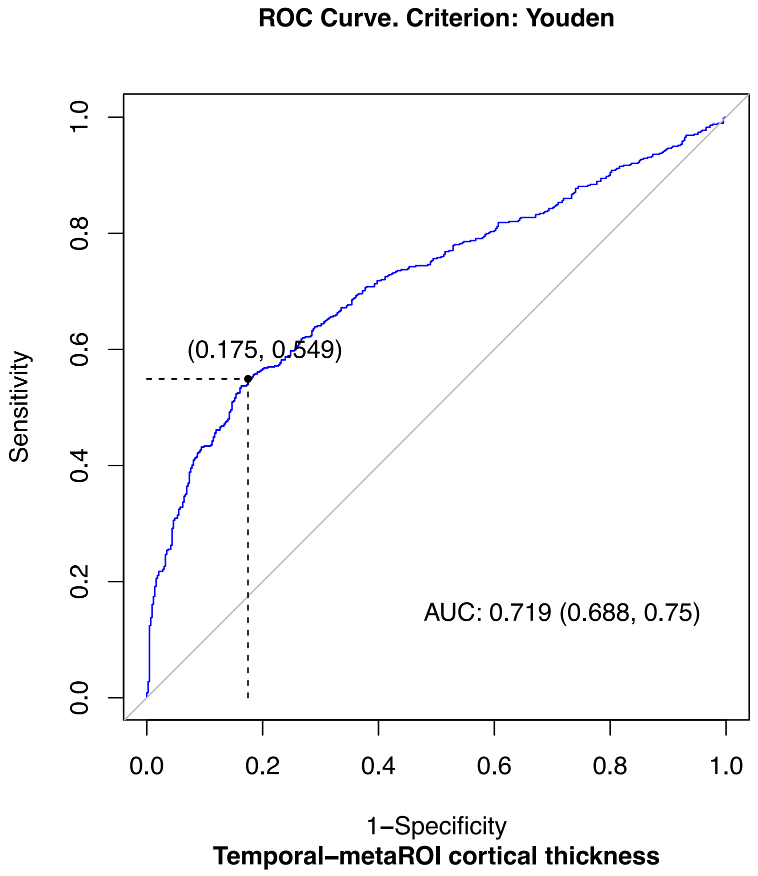

Figure S5. The ROC analysis using cutoff ≥ 2.60 for temporal meta-ROI cortical thickness. The analysis using Youden index classifying 435 Aβ- ADNI CU participants and 579 Aβ+ ADNI MCI and AD patients as the endpoint to define the cutoff ≥2.60 for temporal meta-ROI cortical thickness. AUC: 0.72 [95% ci, 0.688 ~ 0.75].

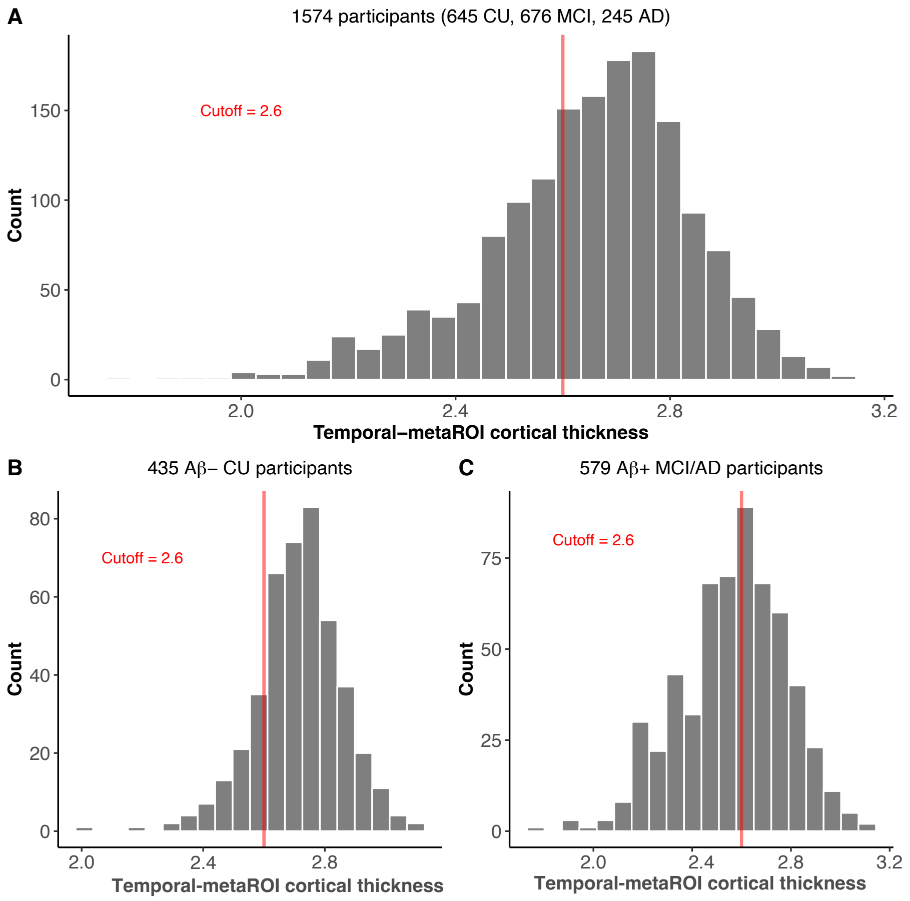

Figure S6. Histograms of temporal meta-ROI cortical thickness within different groups. (A) All 1574 ADNI participants, **(B)** 435 Aβ- ADNI CU participants and **(C)** 579 Aβ+ ADNI MCI and AD patients with MRI data. Red dotted line is the cutoff of temporal meta-ROI cortical thickness (2.60).

Comparisons of baseline and slope of Aβ Centiloids and tau PET in temporal meta-ROI

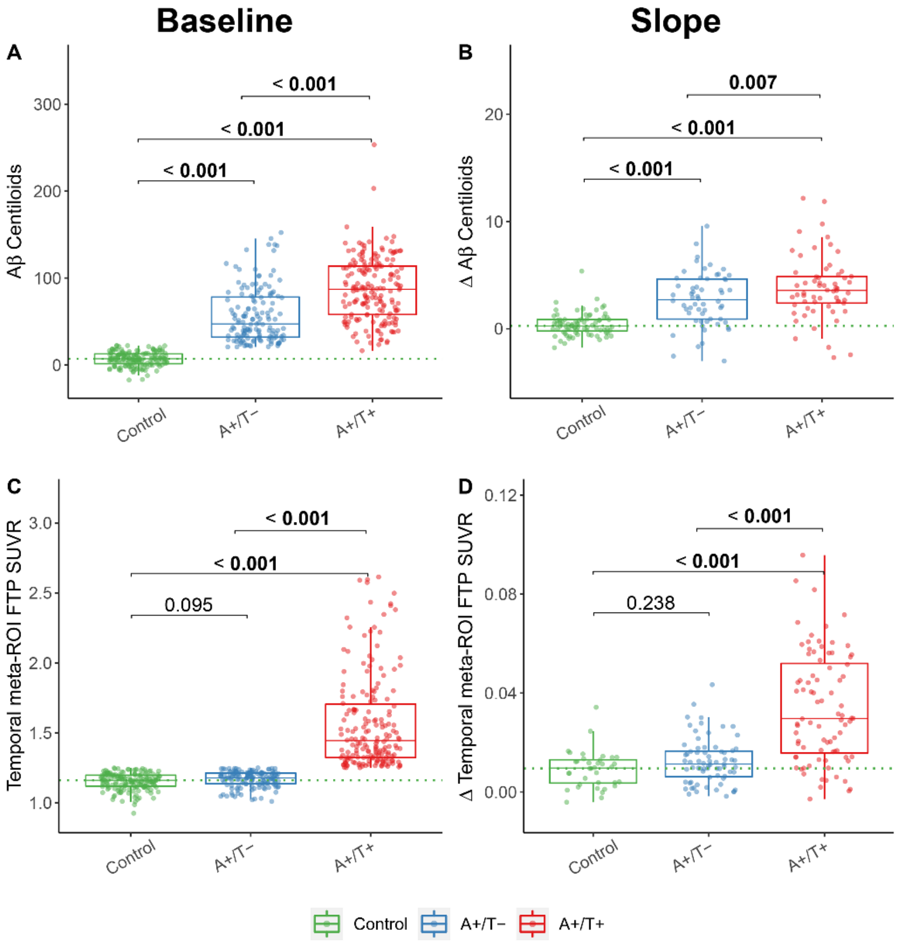

Figure S7. Comparisons of baseline and slope of tau PET in temporal meta-ROI region among the control, A+/T- and A+/T+ groups. Comparisons of baseline and slopes of **(A-B)** Aβ PET Centiloids, **(C-D)** tau PET of temporal meta-ROI region. The boxplot whiskers extended to the lowest and highest data points within 1.5 times the interquartile range from the lower to the upper quartiles. The control, A+/T- and A+/T+ individuals were colored in green, blue and red respectively. Green dashed lines represented the median value of the control group. $P < 0.05$ were marked in bold.

As we defined, while both A+/T- and A+/T+ groups had higher Aβ Centiloids (standardized β (β_{std}) = 1.087, [95% confidence interval (ci), 0.936 ~ 1.237], p < 0.001; β_{std} = 1.781, [95% ci, 1.639 ~ 1.923], p < 0.001) and faster rates of Aβ Centiloids (β_{std} = 0.875, [95% ci, 0.593 ~ 1.157], p < 0.001; β_{std} = 1.291, [95% ci, 1.013 ~ 1.568], p < 0.001) than the control group, A+/T+ but not A+/T- individuals had higher temporal meta-ROI FTP SUVR (β_{std} = 1.534, [95% ci, 1.374 ~ 1.693], p < 0.001) and faster rates of temporal meta-ROI FTP SUVR (β_{std} = 1.285, [95% ci, 0.968 ~ 1.602], p < 0.001) compared to control group (Table S1 and Figure S7). Besides, A+/T+ individuals also had higher baseline (β_{std} = 0.694, [95% ci, 0.554 ~ 0.834], p < 0.001) and slope of Aβ Centiloids (β_{std} = 0.416, [95% ci, 0.116 ~ 0.715], p = 0.007), and higher baseline (β_{std} = 1.389, [95% ci, 1.231 ~ 1.547], p < 0.001) and slope of temporal meta-ROI FTP SUVR (β_{std} = 1.089, [95% ci 0.830 ~ 1.348], p < 0.001) than A+/T- individuals (Table S1 and Figure S7).

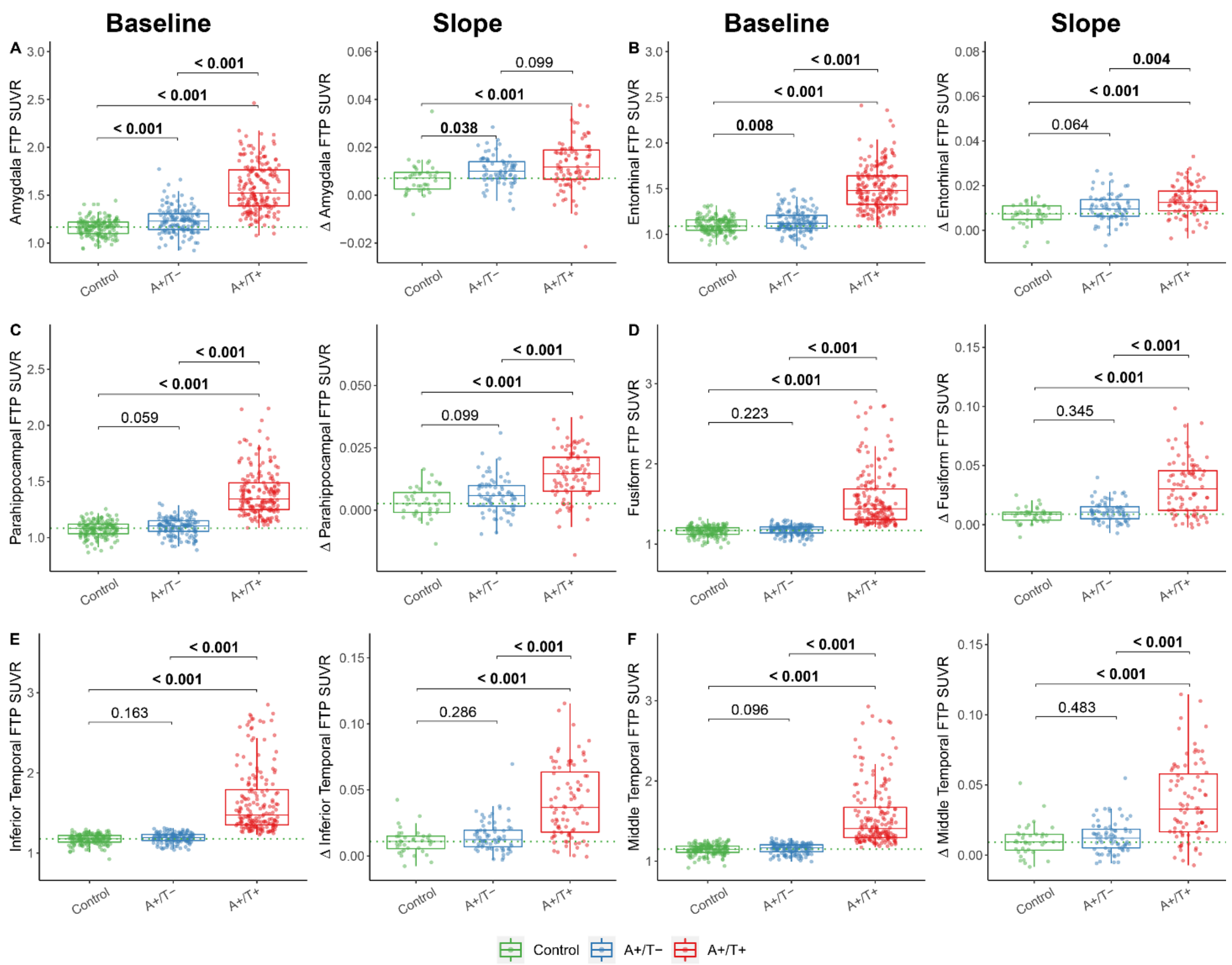

Figure S8. Comparisons of baseline and slopes of tau PET of individual ROIs within temporal meta-ROI among the control, A+/T- and A+/T+ groups. Comparisons of baseline and slopes of tau PET in **(A)** amygdala, **(B)** entorhinal, **(C)** parahippocampal, **(D)** fusiform, **(E)** inferior temporal and **(F)** middle temporal regions. The boxplot whiskers extended to the lowest and highest data points within 1.5 times the interquartile range from the lower to the upper quartiles. The control, A+/T- and A+/T+ individuals were colored in green, blue and red respectively. Green dashed lines represented the median value of the control group. *P* < 0.05 were marked in bold.

Table S1. Comparisons of baseline and slopes of Aβ, regional tau PET of temporal meta-ROI.

		Control vs. A+/T-	Control vs. A+/T+	A+/T- vs. A+/T+			Control vs. A+/T-	Control vs. A+/T+	A+/T- vs. A+/T+
Aβ	β_{std}	1.087	1.781	0.694	Parahip. tau	β_{std}	0.153	1.625	1.472
	95% ci	0.936~1.237	1.639~1.923	0.554~0.834		95% ci	-0.006~0.311	1.475~1.774	1.324~1.620
	p	**< 0.001**	**< 0.001**	**< 0.001**		p	0.059	**< 0.001**	**< 0.001**
Δ Aβ	β_{std}	0.875	1.291	0.416	Δ Parahip. tau	β_{std}	0.296	1.142	0.847
	95% ci	0.593~1.157	1.013~1.568	0.116~0.715		95% ci	-0.054~0.646	0.802~1.483	0.569~1.125
	p	**< 0.001**	**< 0.001**	**0.007**		p	0.099	**< 0.001**	**< 0.001**
Temporal meta-ROI tau	β_{std}	0.144	1.534	1.389	Fusi. tau	β_{std}	0.110	1.438	1.327
	95% ci	-0.025~0.314	1.374~1.693	1.231~1.547		95% ci	-0.067~0.288	1.270~1.605	1.162~1.493
	p	0.095	**< 0.001**	**< 0.001**		p	0.223	**< 0.001**	**< 0.001**
Δ Temporal meta-ROI tau	β_{std}	0.197	1.285	1.089	Δ Fusi. tau	β_{std}	0.160	1.220	1.059
	95% ci	-0.129~0.522	0.968~1.602	0.830~1.348		95% ci	-0.171~0.492	0.897~1.543	0.796~1.323
	p	0.238	**< 0.001**	**< 0.001**		p	0.345	**< 0.001**	**< 0.001**
Amygd. tau	β_{std}	0.276	1.617	1.341	Inf-temp. tau	β_{std}	0.124	1.477	1.352
	95% ci	0.113~0.439	1.463~1.770	0.312~0.392		95% ci	-0.050~0.299	1.312~1.641	1.190~1.515
	p	**< 0.001**	**< 0.001**	**< 0.001**		p	0.163	**< 0.001**	**< 0.001**
Δ Amygd. tau	β_{std}	0.414	0.674	0.260	Δ Inf-temp. tau	β_{std}	0.179	1.264	1.085
	95% ci	0.027~0.801	0.297~1.050	-0.048~0.568		95% ci	-0.149~0.506	0.945~1.582	0.825~1.345
	p	**0.038**	**< 0.001**	0.099		p	0.286	**< 0.001**	**< 0.001**
Entorh. tau	β_{std}	0.212	1.662	1.450	Mid-temp. tau	β_{std}	0.152	1.448	1.296
	95% ci	0.056~0.367	1.515~1.809	1.305~1.595		95% ci	-0.027~0.330	1.279~1.616	1.130~1.463
	p	**0.008**	**< 0.001**	**< 0.001**		p	0.096	**< 0.001**	**< 0.001**
Δ Entorh. tau	β_{std}	0.361	0.810	0.449	Δ Mid-temp. tau	β_{std}	0.121	1.156	1.035
	95% ci	-0.018~0.741	0.441~1.179	0.147~0.750		95% ci	-0.216~0.458	0.828~1.484	0.768~1.303
	p	0.064	**< 0.001**	**0.004**		p	0.483	**< 0.001**	**< 0.001**

Aβ: amyloid-β; Δ: longitudinal tau increase; Amygd.: amygdala; Entorh.: entorhinal; Parahip.: parahippocampal; Fusi.: fusiform; Inf-temp.: inferior temporal; Mid-temp.: middle temporal.
$P < 0.05$ was indicated in bold.

Table S2. Interregional partial correlations of baseline FTP SUVRs in 6 ROIs within temporal meta-ROI region.

		Entorh. tau	Amygd. tau	Parahip. tau	Fusi. tau	Inf-temp. tau	Mid-temp. tau
Entorh. tau	*Rho*	1.000	0.546	0.452	-0.158	0.240	-0.188
	p	1.000	**< 0.001**	**< 0.001**	0.222	**0.001**	**0.039**
Amygd. tau	*Rho*	0.546	1.000	0.269	-0.119	-0.166	0.170
	p	**< 0.001**	1.000	**< 0.001**	1.000	0.142	0.112
Parahip. tau	*Rho*	0.452	0.269	1.000	0.461	0.047	-0.003
	p	**< 0.001**	**< 0.001**	1.000	**< 0.001**	1.000	1.000
Fusi. tau	*Rho*	-0.158	-0.119	0.461	1.000	0.562	-0.203
	p	0.222	1.000	**< 0.001**	1.000	**< 0.001**	**0.015**
Inf-temp. tau	*Rho*	0.240	-0.166	0.047	0.562	1.000	0.823
	p	**0.001**	0.142	1.000	**< 0.001**	1.000	**< 0.001**
Mid-temp. tau	*Rho*	-0.188	0.170	-0.003	-0.203	0.823	1.000
	p	**0.039**	0.112	1.000	**0.015**	**< 0.001**	1.000

Δ: longitudinal tau increase; Amygd.: amygdala; Entorh.: entorhinal; Parahip.: parahippocampal; Fusi.: fusiform; Inf-temp.: inferior temporal; Mid-temp.: middle temporal.
Bonferroni-corrected for multiple comparisons at $p < 0.05$, and $p < 0.05$ was indicated in bold.
Rho: correlation coefficients.

Table S3. Interregional partial correlations of longitudinal FTP SUVR changes in 6 ROIs within temporal meta-ROI region.

		Δ Entorh. tau	Δ Amygd. tau	Δ Parahip. tau	Δ Fusi. tau	Δ Inf-temp. tau	Δ Mid-temp. tau
Δ Entorh. tau	*Rho*	1.000	0.091	0.547	-0.170	0.097	-0.062
	p	1.000	1.000	**< 0.001**	0.109	1.000	1.000
Δ Amygd. tau	*Rho*	0.091	1.000	0.380	0.114	-0.154	-0.020
	p	1.000	1.000	**< 0.001**	1.000	0.270	1.000
Δ Parahip. tau	*Rho*	0.547	0.380	1.000	0.240	0.081	0.065
	p	**< 0.001**	**< 0.001**	1.000	**0.001**	1.000	1.000
Δ Fusi. tau	*Rho*	-0.170	0.114	0.240	1.000	0.544	-0.064
	p	0.109	1.000	**0.001**	1.000	**< 0.001**	1.000
Δ Inf-temp. tau	*Rho*	0.097	-0.154	0.081	0.544	1.000	0.791
	p	1.000	0.270	1.000	**< 0.001**	1.000	**< 0.001**
Δ Mid-temp. tau	*Rho*	-0.062	-0.020	0.065	-0.064	0.791	1.000
	p	1.000	1.000	1.000	1.000	**< 0.001**	1.000

Δ: longitudinal tau increase; Amygd.: amygdala; Entorh.: entorhinal; Parahip.: parahippocampal; Fusi.: fusiform; Inf-temp.: inferior temporal; Mid-temp.: middle temporal.
Bonferroni-corrected for multiple comparisons at $p < 0.05$, and $p < 0.05$ was indicated in bold.
Rho: correlation coefficients.

Table S4. Longitudinal tau accumulation prediction by baseline amygdala tau or entorhinal tau adjusted for baseline Aβ Centiloids.

		Aβ + Amygd. tau				Aβ + Entorh. tau			
		A+/T-		A+/T+		A+/T-		A+/T+	
		Aβ	Amygd. tau	Aβ	Amygd. tau	Aβ	Entorh. tau	Aβ	Entorh. tau
Δ Amygd. tau	β_{std}	0.241	0.509	0.051	0.485	0.192	0.577	0.044	0.452
	95% ci	0.045~0.437	0.313~0.704	-0.144~0.245	0.290~0.680	0.006~0.378	0.391~0.763	-0.157~0.245	0.251~0.652
	p	**0.019**	**< 0.001**	0.612	**< 0.001**	**0.047**	**< 0.001**	0.671	**< 0.001**
Δ Entorh. tau	β_{std}	0.294	0.080	0.184	0.092	0.272	0.233	0.128	0.279
	95% ci	0.068~0.520	-0.146~0.306	-0.035~0.403	-0.127~0.311	0.050~0.493	0.012~0.455	-0.086~0.341	0.065~0.493
	p	**0.013**	0.491	0.104	0.414	**0.019**	**0.043**	0.245	**0.012**
Δ Parahip. tau	β_{std}	0.314	0.088	0.156	0.306	0.284	0.309	0.100	0.471
	95% ci	0.089~0.538	-0.136~0.313	-0.052~0.365	0.098~0.515	0.070~0.499	0.094~0.523	-0.096~0.295	0.275~0.666
	p	**0.008**	0.444	0.145	**0.005**	**0.012**	**0.006**	0.322	**< 0.001**
Δ Fusi. tau	β_{std}	0.353	-0.045	0.337	0.259	0.337	0.142	0.240	0.569
	95% ci	0.130~0.575	-0.267~0.178	0.138~0.535	0.060~0.457	0.116~0.558	-0.079~0.363	0.073~0.407	0.402~0.736
	p	**0.003**	0.696	**0.002**	**0.012**	**0.004**	0.211	**0.006**	**< 0.001**
Δ Inf-temp. tau	β_{std}	0.179	-0.113	0.284	0.261	0.173	0.041	0.180	0.597
	95% ci	-0.053~0.412	-0.345~0.119	0.081~0.487	0.058~0.464	-0.062~0.408	-0.194~0.276	0.011~0.348	0.428~0.765
	p	0.135	0.344	**0.008**	**0.014**	0.153	0.733	**0.040**	**< 0.001**
Δ Mid-temp. tau	β_{std}	0.095	-0.060	0.282	0.282	0.082	0.112	0.186	0.585
	95% ci	-0.142~0.331	-0.297~0.176	0.080~0.483	0.080~0.483	-0.154~0.319	-0.125~0.348	0.016~0.356	0.415~0.755
	p	0.436	0.618	**0.008**	**0.008**	0.497	0.358	**0.035**	**< 0.001**

Δ: longitudinal tau increase; Amygd.: amygdala; Entorh.: entorhinal; Parahip.: parahippocampal; Fusi.: fusiform; Inf-temp.: inferior temporal; Mid-temp.: middle temporal.
P < 0.05 was indicated in bold.

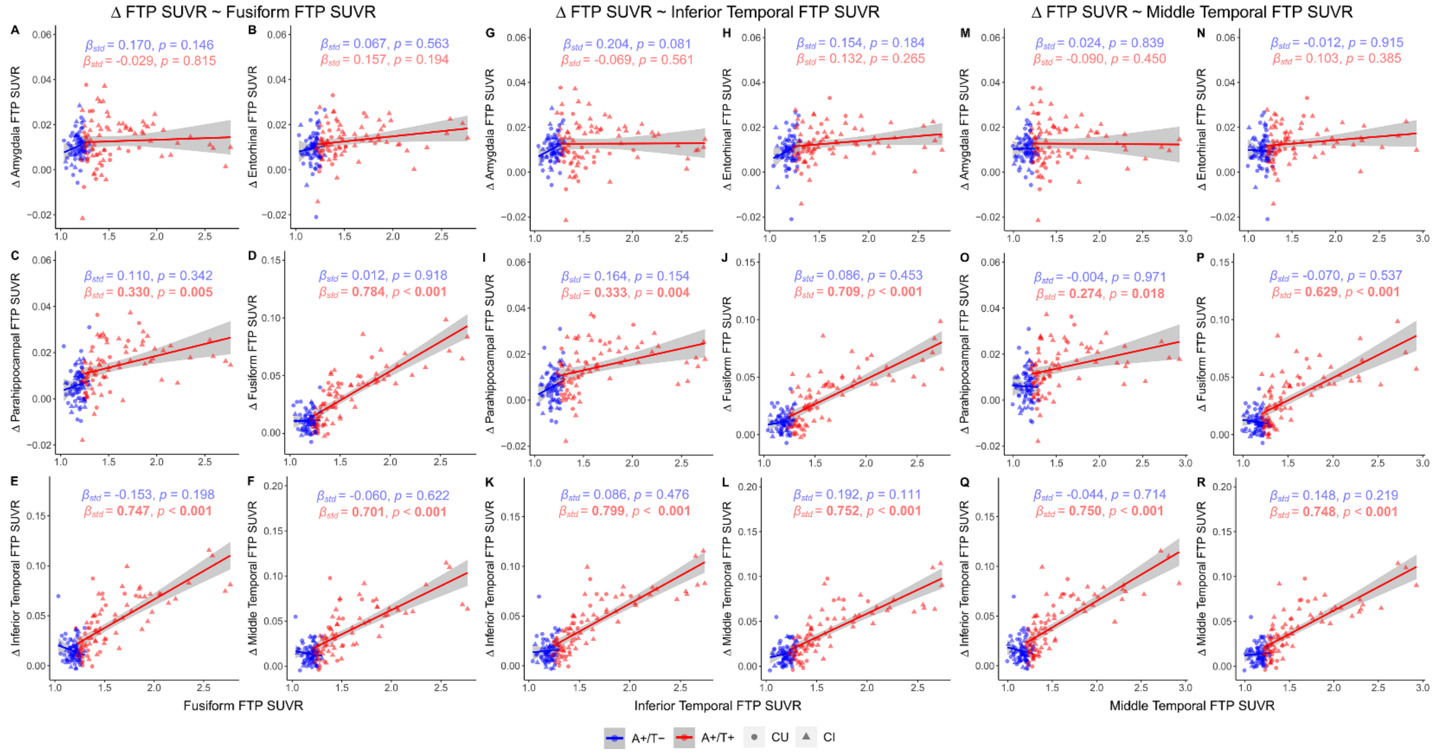

Figure S9. Prediction of longitudinal tau accumulation by baseline tau in fusiform, inferior temporal or middle temporal regions. Models have been accounted for age, sex and baseline Aβ Centiloids. Prediction of longitudinal FTP SUVR increases in **(A)** amygdala, **(B)** entorhinal, **(C)** parahippocampal, **(D)** fusiform, **(E)** inferior temporal and **(F)** middle temporal regions by baseline fusiform FTP SUVR. Prediction of longitudinal FTP SUVR increases in **(G)** amygdala, **(H)** entorhinal, **(I)** parahippocampal, **(J)** fusiform, **(K)** inferior temporal and **(L)** middle temporal regions by baseline inferior temporal FTP SUVR. Prediction of longitudinal FTP SUVR increases in **(M)** amygdala, **(N)** entorhinal, **(O)** parahippocampal, **(P)** fusiform, **(Q)** inferior temporal and **(R)** middle temporal regions by baseline middle temporal FTP SUVR. Linear regression lines, each individual point, β_{std} and p values of A+/T- and A+/T+ groups were colored in blue and red, and CU and CI individuals were represented by circle and triangle respectively. Linear model fits were indicated with 95% confidence intervals. $P < 0.05$ and associated β_{std} were marked in bold.

Table S5. Longitudinal tau accumulation prediction by baseline parahippocampal tau or fusiform tau adjusted for Aβ Centiloids.

		Aβ + Parahip. tau				Aβ + Fusi. tau			
		A+/T-		A+/T+		A+/T-		A+/T+	
		Aβ	Parahip. tau	Aβ	Parahip. tau	Aβ	Fusi. tau	Aβ	Fusi. tau
Δ Amygd. tau	β_{std}	0.258	0.294	0.118	0.148	0.247	0.170	0.184	-0.029
	95% ci	0.039~0.478	0.075~0.513	-0.110~0.346	-0.080~0.376	0.020~0.473	-0.056~0.397	-0.055~0.423	-0.267~0.210
	p	**0.024**	**0.011**	0.315	0.206	**0.036**	0.146	0.135	0.815
Δ Entorh. tau	β_{std}	0.299	0.119	0.116	0.250	0.294	0.067	0.138	0.157
	95% ci	0.073~0.524	-0.106~0.345	-0.106~0.338	0.028~0.472	0.067~0.521	-0.159~0.294	-0.097~0.372	-0.078~0.392
	p	**0.012**	0.303	0.309	**0.030**	**0.013**	0.563	0.254	0.194
Δ Parahip. tau	β_{std}	0.321	0.200	0.045	0.519	0.313	0.110	0.087	0.330
	95% ci	0.100~0.541	-0.020~0.421	-0.153~0.242	0.322~0.716	0.089~0.537	-0.115~0.334	-0.137~0.312	0.106~0.555
	p	**0.006**	0.080	0.660	< **0.001**	**0.008**	0.342	0.448	**0.005**
Δ Fusi. tau	β_{std}	0.352	0.003	0.138	0.724	0.352	0.012	0.056	0.784
	95% ci	0.129~0.574	-0.219~0.226	-0.007~0.283	0.580~0.869	0.129~0.574	-0.211~0.234	-0.086~0.198	0.642~0.926
	p	**0.003**	0.976	0.065	< **0.001**	**0.003**	0.918	0.441	< **0.001**
Δ Inf-temp. tau	β_{std}	0.172	-0.188	0.074	0.758	0.180	-0.153	0.020	0.747
	95% ci	-0.058~0.402	-0.418~0.042	-0.070~0.218	0.614~0.902	-0.051~0.411	-0.384~0.078	-0.139~0.179	0.588~0.906
	p	0.147	0.113	0.319	< **0.001**	0.131	0.198	0.806	< **0.001**
Δ Mid-temp. tau	β_{std}	0.091	-0.091	0.082	0.745	0.095	-0.060	0.044	0.701
	95% ci	-0.145~0.327	-0.327~0.145	-0.065~0.228	0.598~0.892	-0.142~0.331	-0.296~0.177	-0.125~0.212	0.533~0.869
	p	0.452	0.452	0.280	< **0.001**	0.435	0.622	0.613	< **0.001**

Δ: longitudinal tau increase; Amygd.: amygdala; Entorh.: entorhinal; Parahip.: parahippocampal; Fusi.: fusiform; Inf-temp.: inferior temporal; Mid-temp.: middle temporal.
$P < 0.05$ was indicated in bold.

Table S6. Longitudinal tau accumulation prediction by baseline inferior temporal tau or middle temporal tau adjusted for Aβ Centiloids.

		Aβ + Inf-temp. tau				Aβ + Mid-temp. tau			
		A+/T-		A+/T+		A+/T-		A+/T+	
		Aβ	Inf-temp. tau	Aβ	Inf-temp. tau	Aβ	Mid-temp. tau	Aβ	Mid-temp. tau
Δ Amygd. tau	β_{std}	0.230	0.204	0.198	-0.069	0.251	0.024	0.206	-0.090
	95% ci	0.004~0.456	-0.022~0.431	-0.034~0.431	-0.302~0.163	0.021~0.482	-0.206~0.254	-0.026~0.438	-0.322~0.142
	p	**0.050**	0.081	0.098	0.561	**0.036**	0.839	0.085	0.450
Δ Entorh. tau	β_{std}	0.280	0.154	0.155	0.132	0.295	-0.012	0.167	0.103
	95% ci	0.055~0.505	-0.071~0.379	-0.074~0.385	-0.098~0.361	0.067~0.522	-0.240~0.215	-0.063~0.397	-0.128~0.333
	p	**0.017**	0.184	0.188	0.265	**0.013**	0.915	0.160	0.385
Δ Parahip. tau	β_{std}	0.299	0.164	0.103	0.333	0.315	-0.004	0.126	0.274
	95% ci	0.076~0.522	-0.059~0.388	-0.115~0.321	0.115~0.551	0.089~0.541	-0.230~0.222	-0.096~0.348	0.052~0.496
	p	**0.011**	0.154	0.357	**0.004**	**0.008**	0.971	0.269	**0.018**
Δ Fusi. tau	β_{std}	0.343	0.086	0.125	0.709	0.348	-0.070	0.157	0.629
	95% ci	0.121~0.566	-0.137~0.308	-0.027~0.277	0.557~0.861	0.126~0.571	-0.292~0.152	-0.011~0.324	0.461~0.797
	p	**0.004**	0.453	0.110	**< 0.001**	**0.003**	0.537	0.071	**< 0.001**
Δ Inf-temp. tau	β_{std}	0.169	0.086	0.038	0.799	0.175	-0.044	0.057	0.750
	95% ci	-0.065~0.403	-0.149~0.320	-0.099~0.175	0.662~0.936	-0.059~0.409	-0.278~0.190	-0.092~0.207	0.601~0.900
	p	0.162	0.476	0.591	**< 0.001**	0.147	0.714	0.455	**< 0.001**
Δ Mid-temp. tau	β_{std}	0.075	0.192	0.059	0.752	0.101	0.148	0.061	0.748
	95% ci	-0.159~0.308	-0.041~0.426	-0.090~0.208	0.604~0.901	-0.134~0.335	-0.086~0.383	-0.088~0.211	0.598~0.897
	p	0.533	0.111	0.438	**< 0.001**	0.403	0.219	0.424	**< 0.001**

Δ: longitudinal tau increase; Amygd.: amygdala; Entorh.: entorhinal; Parahip.: parahippocampal; Fusi.: fusiform; Inf-temp.: inferior temporal; Mid-temp.: middle temporal.
P < 0.05 was indicated in bold.

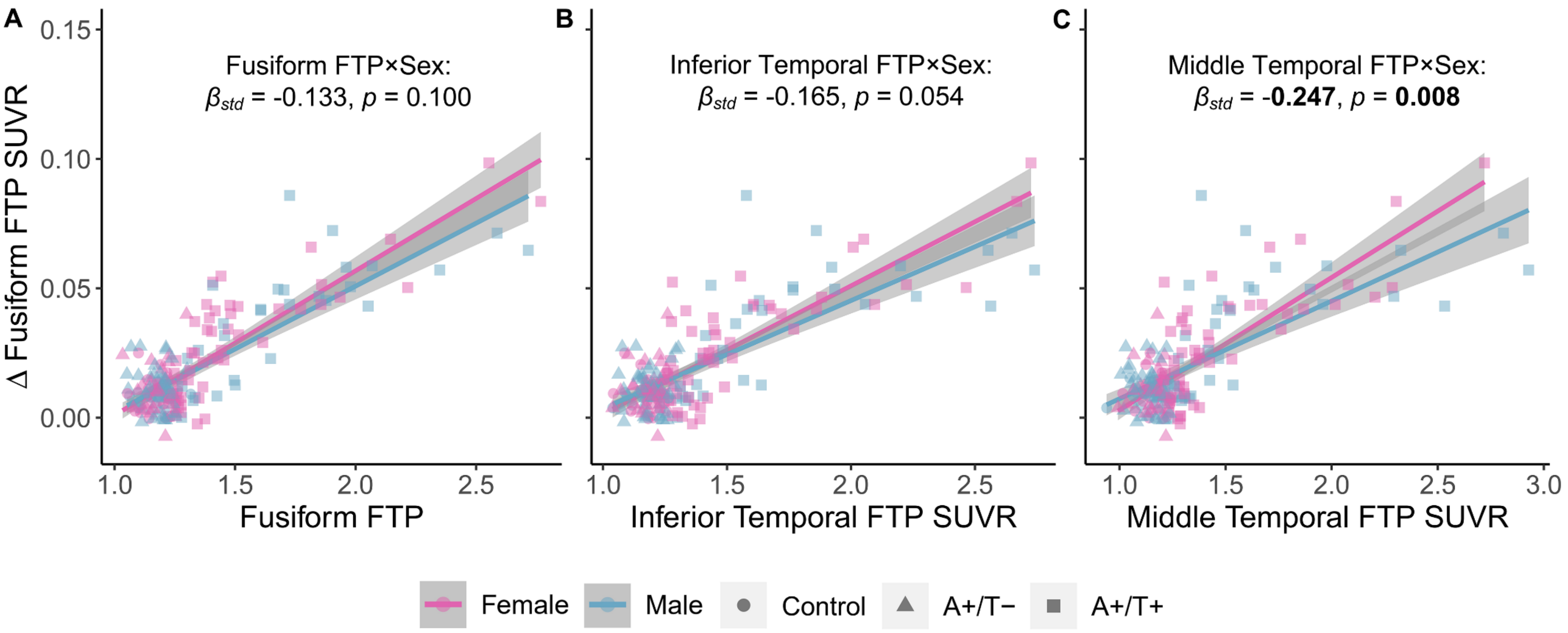

Figure S10. Aβ and tau related longitudinal tau accumulation in female and male adults. Positive interactions of **(A)** female × fusiform FTP SUVR, **(B)** female × inferior temporal FTP SUVR, and **(C)** female × middle temporal FTP SUVR on longitudinal tau accumulations in fusiform. Linear regression lines and each individual point of females and males were colored in violet and pacific blue, and the control, A+/T- and A+/T+ groups were represented by circle, triangle and square respectively. Linear model fits were indicated with 95% confidence intervals. $P < 0.05$ and associated β_{std} were marked in bold.

Table S7. Prediction of longitudinal tau accumulation by interactions of sex and baseline Aβ/tau level.

			Δ Amygd. tau	Δ Entorh. tau	Δ Parahip. tau	Δ Fusi. tau	Δ Inf-temp. tau	Δ Mid-temp. tau
Sex (male Vs. female)	*Aβ×Sex*	β_{std}	0.158	-0.031	0.054	0.168	0.198	0.212
		95% ci	-0.115~0.431	-0.298~0.237	-0.203~0.310	-0.076~0.412	-0.051~0.448	-0.043~0.466
		p	0.259	0.822	0.683	0.178	0.121	0.105
	Aβ+*Amygd. tau×Sex*	β_{std}	0.076	0.034	0.007	0.048	0.046	0.050
		95% ci	-0.177~0.328	-0.233~0.302	-0.237~0.250	-0.182~0.278	-0.189~0.282	-0.189~0.289
		p	0.557	0.802	0.958	0.682	0.699	0.681
	Aβ+*Entorh. tau×Sex*	β_{std}	0.087	0.022	-0.010	0.052	0.087	0.092
		95% ci	-0.170~0.344	-0.240~0.284	-0.238~0.217	-0.147~0.251	-0.116~0.291	-0.116~0.301
		p	0.509	0.870	0.929	0.609	0.401	0.387
	Aβ+*Parahip. tau×Sex*	β_{std}	0.110	0.090	-0.009	-0.038	0.017	0.024
		95% ci	-0.161~0.381	-0.172~0.351	-0.235~0.217	-0.212~0.136	-0.164~0.198	-0.162~0.210
		p	0.426	0.501	0.936	0.665	0.858	0.802
	Aβ+*Fusi. tau×Sex*	β_{std}	0.057	0.123	-0.067	-0.133	-0.007	0.007
		95% ci	-0.218~0.332	-0.143~0.388	-0.309~0.175	-0.290~0.025	-0.185~0.171	-0.181~0.195
		p	0.684	0.365	0.589	0.100	0.938	0.944
	Aβ+*Inf-temp. tau×Sex*	β_{std}	0.036	0.062	-0.127	-0.165	-0.058	-0.055
		95% ci	-0.239~0.311	-0.204~0.329	-0.367~0.113	-0.331~0.002	-0.220~0.104	-0.228~0.119
		p	0.798	0.648	0.301	0.054	0.484	0.536
	Aβ+*Mid-temp. tau×Sex*	β_{std}	0.009	0.026	-0.188	-0.247	-0.133	-0.144
		95% ci	-0.268~0.287	-0.244~0.296	-0.434~0.058	-0.428~-0.065	-0.306~0.041	-0.319~0.030
		p	0.947	0.849	0.135	**0.008**	0.137	0.107

Δ: longitudinal tau increase; Amygd.: amygdala; Entorh.: entorhinal; Parahip.: parahippocampal; Fusi.: fusiform; Inf-temp.: inferior temporal; Mid-temp.: middle temporal. Interaction term was marked in italic, and $p < 0.05$ was indicated in bold.

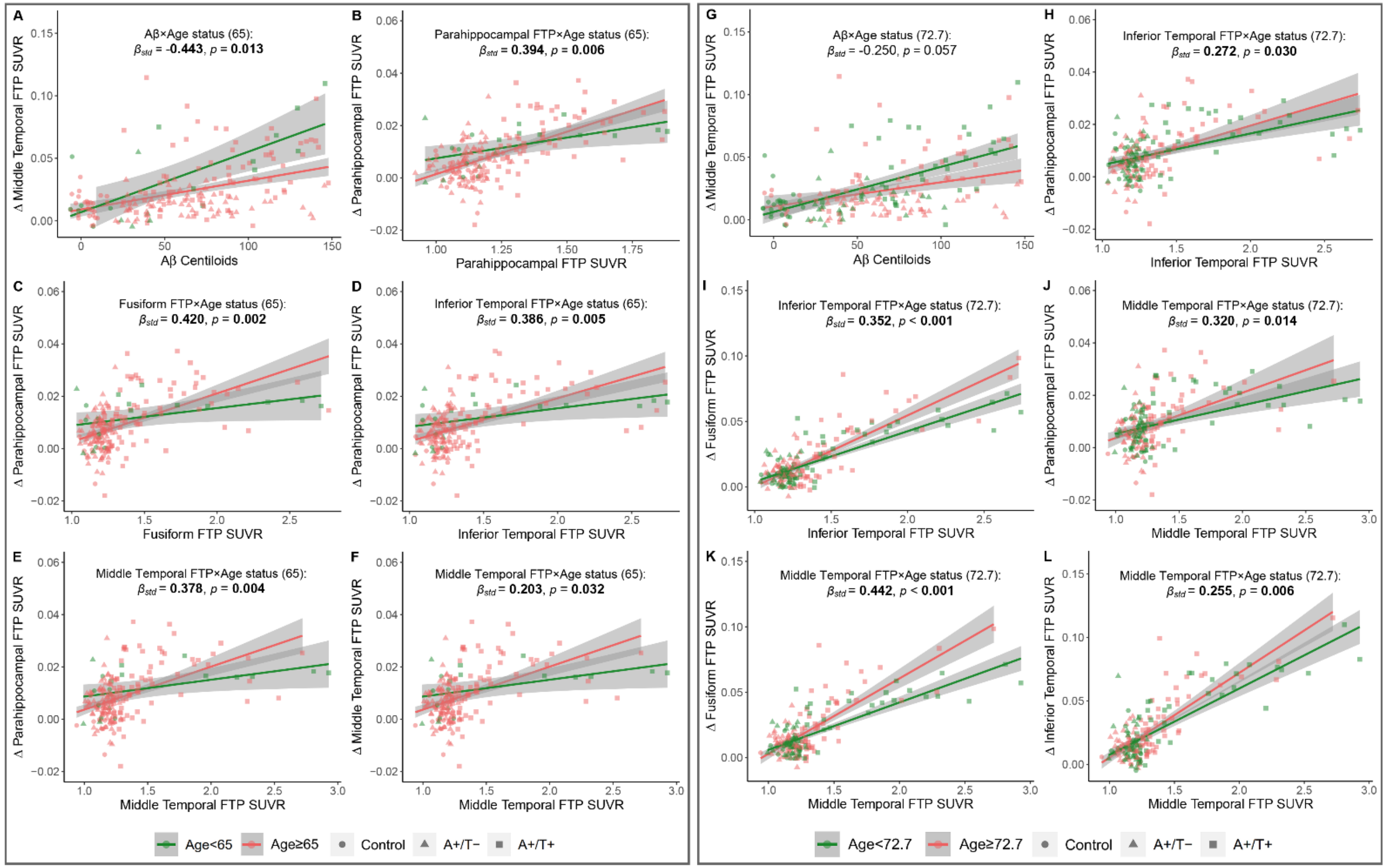

Figure S11. Aβ and tau related longitudinal tau accumulation in early-life and late-life elderly adults. Results based on cut age of 65 year and median age (72.7 year) were depicted respectively in left **(A-F)** and right **(G-H)** panels. Linear regression lines and each individual point of early-life elderly adults (age<65 and age<72.7 years) and late-life elderly adults (age⩾65 and age⩾72.7 years) were colored in salmon and green, and the control, A+/T- and A+/T+ groups were represented by circle, triangle and square respectively. Linear model fits were indicated with 95% confidence intervals. $P < 0.05$ and associated β_{std} were marked in bold.

Table S8. Prediction of longitudinal tau accumulation by interactions of age status (cut age of 65 year) and baseline Aβ/tau level.

			Δ Amygd. tau	Δ Entorh. tau	Δ Parahip. tau	Δ Fusi. tau	Δ Inf-temp. tau	Δ Mid-temp. tau
Age status (65) (age≥65 Vs. age<65)	*Aβ×Age status*	β_{std}	0.145	-0.001	0.124	-0.329	-0.374	-0.443
		95% ci	-0.237~0.526	-0.374~0.372	-0.232~0.481	-0.662~0.005	-0.714~-0.034	-0.790~-0.096
		p	0.458	0.995	0.495	0.055	**0.032**	**0.013**
	Aβ+*Amygd. Tau×Age status*	β_{std}	0.251	0.063	0.212	0.010	-0.019	-0.179
		95% ci	-0.065~0.566	-0.273~0.399	-0.091~0.516	-0.275~0.295	-0.310~0.273	-0.475~0.117
		p	0.121	0.713	0.172	0.946	0.899	0.238
	Aβ+*Entorh. Tau×Age status*	β_{std}	0.192	0.097	0.263	0.002	-0.036	-0.169
		95% ci	-0.158~0.543	-0.261~0.454	-0.044~0.570	-0.267~0.271	-0.310~0.239	-0.452~0.114
		p	0.283	0.597	0.095	0.989	0.798	0.243
	Aβ+*Parahip. Tau×Age status*	β_{std}	0.267	0.117	0.394	0.158	0.097	-0.063
		95% ci	-0.071~0.604	-0.211~0.445	0.117~0.671	-0.057~0.374	-0.127~0.321	-0.295~0.169
		p	0.124	0.484	**0.006**	0.152	0.398	0.596
	Aβ+*Fusi. Tau×Age status*	β_{std}	0.316	0.086	0.420	0.338	0.257	0.140
		95% ci	0.015~0.618	-0.209~0.382	0.157~0.682	0.168~0.507	0.063~0.451	-0.068~0.348
		p	**0.041**	0.567	**0.002**	**< 0.001**	**0.010**	0.189
	Aβ+*Inf-temp. tau×Age status*	β_{std}	0.232	0.084	0.386	0.292	0.233	0.110
		95% ci	-0.076~0.541	-0.216~0.385	0.120~0.652	0.107~0.477	0.054~0.413	-0.086~0.305
		p	0.141	0.583	**0.005**	**0.002**	**0.012**	0.273
	Aβ+*Mid-temp. tau×Age status*	β_{std}	0.178	0.084	0.378	0.350	0.322	0.203
		95% ci	-0.114~0.469	-0.201~0.369	0.121~0.634	0.160~0.540	0.143~0.502	0.018~0.388
		p	0.234	0.565	**0.004**	**< 0.001**	**0.001**	**0.032**

Δ: longitudinal tau increase; Amygd.: amygdala; Entorh.: entorhinal; Parahip.: parahippocampal; Fusi.: fusiform; Inf-temp.: inferior temporal; Mid-temp.: middle temporal.
Interaction term was marked in italic, and $p < 0.05$ was indicated in bold.

Table S9. Prediction of longitudinal tau accumulation by interactions of age status (cut age of 72.7 year) and baseline Aβ/tau level.

			Δ Amygd. Tau	Δ Entorh. Tau	Δ Parahip. Tau	Δ Fusi. Tau	Δ Inf-temp. tau	Δ Mid-temp. tau
Age status (72.7) (age≥72.7 Vs. age<72.7)	Aβ×*Age status*	β_{std}	0.161	0.162	0.144	-0.086	-0.233	-0.250
		95% ci	-0.114~0.436	-0.108~0.432	-0.116~0.403	-0.334~0.162	-0.484~0.019	-0.507~0.006
		p	0.252	0.241	0.279	0.499	0.071	0.057
	Aβ+*Amygd. Tau×Age status*	β_{std}	0.089	0.042	0.118	0.158	0.030	-0.033
		95% ci	-0.164~0.343	-0.228~0.311	-0.127~0.363	-0.073~0.389	-0.206~0.267	-0.274~0.208
		p	0.492	0.761	0.347	0.182	0.802	0.790
	Aβ+*Entorh. Tau×Age status*	β_{std}	0.067	0.153	0.228	0.264	0.107	0.065
		95% ci	-0.192~0.326	-0.111~0.417	0.001~0.456	0.066~0.462	-0.098~0.312	-0.146~0.277
		p	0.614	0.258	0.050	**0.010**	0.310	0.547
	Aβ+*Parahip. Tau×Age status*	β_{std}	0.179	0.035	0.158	0.167	-0.004	-0.079
		95% ci	-0.097~0.456	-0.235~0.304	-0.073~0.39	-0.011~0.345	-0.190~0.181	-0.270~0.112
		p	0.205	0.801	0.181	0.067	0.963	0.416
	Aβ+*Fusi. Tau×Age status*	β_{std}	0.247	0.076	0.256	0.267	0.069	-0.012
		95% ci	-0.029~0.522	-0.195~0.346	0.013~0.5	0.110~0.425	-0.112~0.25	-0.204~0.179
		p	0.081	0.584	**0.041**	**0.001**	0.457	0.901
	Aβ+*Inf-temp. tau×Age status*	β_{std}	0.207	0.079	0.272	0.352	0.135	0.058
		95% ci	-0.071~0.484	-0.193~0.350	0.029~0.515	0.187~0.516	-0.030~0.300	-0.120~0.236
		p	0.146	0.571	**0.030**	**< 0.001**	0.110	0.523
	Aβ+*Mid-temp. tau×Age status*	β_{std}	0.166	0.087	0.320	0.442	0.255	0.170
		95% ci	-0.120~0.451	-0.193~0.367	0.066~0.574	0.260~0.624	0.076~0.434	-0.011~0.352
		p	0.257	0.545	**0.014**	**< 0.001**	**0.006**	0.068

Δ: longitudinal tau increase; Amygd.: amygdala; Entorh.: entorhinal; Parahip.: parahippocampal; Fusi.: fusiform; Inf-temp.: inferior temporal; Mid-temp.: middle temporal. Interaction term was marked in italic, and $p < 0.05$ was indicated in bold.

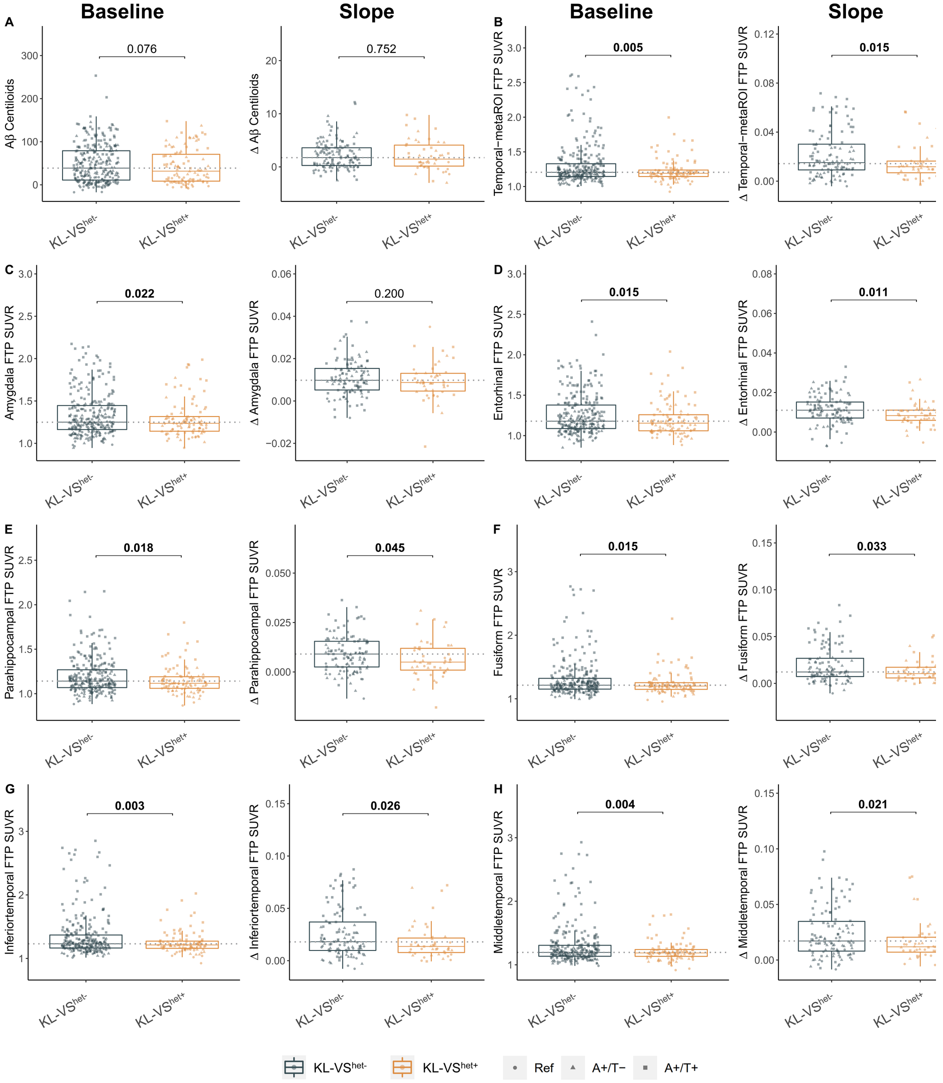

Figure S12. Comparisons of baseline and slopes of Aβ Centiloids and tau PET in temporal meta-ROI region and 6 ROIs within this composite region between KL-VS^{het-} and KL-VS^{het+} groups. Comparisons of baseline and slopes of **(A)** Aβ Centiloids, **(B)** tau PET in temporal meta-ROI region, **(C)** amygdala, **(D)** entorhinal, **(E)** parahippocampal, **(F)** fusiform, **(G)** inferior temporal and **(H)** middle temporal regions. The boxplot whiskers extend to the lowest and highest data points within 1.5 times the interquartile range from the lower to the upper quartiles. KL-VS^{het-} and KL-VS^{het+} individuals were colored in gray and orange, and the control, A+/T- and A+/T+ individuals were represented by circle, triangle and square respectively. Gray dashed lines represented the median value of the KL-VS^{het-} group. $P < 0.05$ were marked in bold.

Table S10. Comparisons of baseline and slopes of Aβ Centiloids, regional tau PET in temporal meta-ROI between KL-VS^{het-} and KL-VS^{het+}.

Aβ	β_{std}	-0.182	Parahip. tau	β_{std}	-0.263
	95% ci	-0.382~0.018		95% ci	-0.481~-0.046
	p	0.076		*p*	**0.018**
Δ Aβ	β_{std}	-0.052	Δ Parahip. tau	β_{std}	-0.340
	95% ci	-0.372~0.269		95% ci	-0.669~-0.011
	p	0.752		*p*	**0.045**
Temporal meta-ROI tau	β_{std}	-0.317	Fusi. tau	β_{std}	-0.276
	95% ci	-0.536~-0.098		95% ci	-0.497~-0.055
	p	**0.005**		*p*	**0.015**
Δ Temporal meta-ROI tau	β_{std}	-0.413	Δ Fusi. tau	β_{std}	-0.362
	95% ci	-0.742~-0.083		95% ci	-0.692~-0.031
	p	**0.015**		*p*	**0.033**
Amygd. tau	β_{std}	-0.241	Inf-temp. tau	β_{std}	-0.330
	95% ci	-0.448~-0.035		95% ci	-0.550~-0.110
	p	**0.022**		*p*	**0.003**
Δ Amygd. tau	β_{std}	-0.216	Δ Inf-temp. tau	β_{std}	-0.379
	95% ci	-0.546~0.113		95% ci	-0.710~-0.047
	p	0.200		*p*	**0.026**
Entorh. tau	β_{std}	-0.263	Mid-temp. tau	β_{std}	-0.328
	95% ci	-0.474~-0.052		95% ci	-0.548~-0.107
	p	**0.015**		*p*	**0.004**
Δ Entorh. tau	β_{std}	-0.430	Δ Mid-temp. tau	β_{std}	-0.396
	95% ci	-0.756~-0.104		95% ci	-0.730~-0.062
	p	**0.011**		*p*	**0.021**

Δ: longitudinal tau increase; Amygd.: amygdala; Entorh.: entorhinal; Parahip.: parahippocampal; Fusi.: fusiform; Inf-temp.: inferior temporal; Mid-temp.: middle temporal.
$P < 0.05$ was indicated in bold.

Table S11. The median slopes of longitudinal tau accumulation in temporal meta-ROI and all individual ROIs in temporal meta-ROI in KL-VS^{het-} and KL-VS^{het+} individuals.

	**KL-VS^{het-} (median slope)**	**KL-VS^{het+} (median slope)**	**KL-VS^{het+}/KL-VS^{het-} ratio**
**Δ Temporal meta-ROI**	0.014	0.012	0.820
**Δ Amygd. tau**	0.010	0.009	0.892
**Δ Entorh.**	0.011	0.008	0.755
**Δ Parahip.**	0.009	0.005	0.551
**Δ Fusi.**	0.012	0.010	0.856
**Δ Inf-temp.**	0.018	0.014	0.757
**Δ Mid-temp.**	0.017	0.012	0.679

Amygd.: amygdala; Entorh.: entorhinal; Parahip.: parahippocampal; Fusi.: fusiform; Inf-temp.: inferior temporal; Mid-temp.: middle temporal.
$P < 0.05$ was indicated in bold.

Table S12. Prediction of longitudinal tau increase by interactions of KL-VS^{het} genotype and baseline Aβ/tau level.

			Δ Amygd. Tau	Δ Entorh. Tau	Δ Parahip. Tau	Δ Fusi. Tau	Δ Inf-temp. tau	Δ Mid-temp. tau
KL_VS^{het} (KL-VS^{het+} Vs. KL-VS^{het-})	*Aβ*×KL-VS^{het}	β_{std}	-0.193	-0.184	-0.359	-0.374	-0.405	-0.366
		95% ci	-0.516~0.129	-0.501~0.133	-0.647~-0.071	-0.622~-0.127	-0.662~-0.148	-0.629~-0.103
		p	0.242	0.257	**0.016**	**0.003**	**0.002**	**0.007**
	Aβ+*Amygd. tau*×KL-VS^{het}	β_{std}	0.050	-0.087	-0.108	-0.185	-0.220	-0.175
		95% ci	-0.299~0.400	-0.450~0.276	-0.431~0.215	-0.450~0.080	-0.500~0.060	-0.459~0.108
		p	0.778	0.639	0.512	0.174	0.125	0.227
	Aβ+*Entorh. tau*×KL-VS^{het}	β_{std}	0.106	-0.028	-0.080	-0.286	-0.289	-0.196
		95% ci	-0.276~0.487	-0.420~0.363	-0.412~0.253	-0.542~-0.029	-0.560~-0.018	-0.476~0.083
		p	0.587	0.887	0.640	**0.030**	**0.038**	0.170
	Aβ+*Parahip. tau*×KL-VS^{het}	β_{std}	0.141	0.050	0.016	-0.222	-0.236	-0.139
		95% ci	-0.303~0.585	-0.386~0.486	-0.355~0.387	-0.482~0.038	-0.520~0.048	-0.433~0.156
		p	0.535	0.823	0.931	0.096	0.106	0.357
	Aβ+*Fusi. tau*×KL-VS^{het}	β_{std}	0.035	0.0751	0.075	-0.100	-0.136	0.007
		95% ci	-0.571~0.641	-0.462~0.612	-0.462~0.612	-0.434~0.233	-0.529~0.257	-0.411~0.425
		p	0.910	0.784	0.784	0.556	0.499	0.974
	Aβ+*Inf-temp. tau*×KL-VS^{het}	β_{std}	0.390	0.162	0.227	-0.045	0.036	0.209
		95% ci	-0.254~1.035	-0.473~0.797	-0.326~0.780	-0.397~0.307	-0.333~0.406	-0.189~0.607
		p	0.237	0.618	0.423	0.804	0.847	0.305
	Aβ+*Mid-temp. tau*×KL-VS^{het}	β_{std}	0.465	0.156	0.202	-0.062	0.028	0.244
		95% ci	-0.184~1.114	-0.485~0.798	-0.366~0.769	-0.454~0.329	-0.368~0.424	-0.156~0.644
		p	0.162	0.634	0.487	0.756	0.889	0.234

Δ: longitudinal tau increase; Amygd.: amygdala; Entorh.: entorhinal; Parahip.: parahippocampal; Fusi.: fusiform; Inf-temp.: inferior temporal; Mid-temp.: middle temporal.
Interaction term was marked in italic, and $p < 0.05$ was indicated in bold.

Sensitivity analysis

Table S13. Demographic Characteristics of Participants with temporal meta-ROI FTP SUVR threshold = 1.27.

	Control (A-/T-/N-/CU)	A+/T-	A+/T+
Participants with concurrent Aβ PET, tau PET and MRI at baseline (n = 487)			
A/T/N, *n* (%)	162 (33.3)	158 (32.4)	167 (34.3)
Diagnosis, CU:CI	162:0	100:58	41:126
Females, *n* (%)	102 (63)	76 (48.1)[a]	88 (52.7)
Age, years, median (IQR, range)	69.2 (9.3, 62.8-82.7)	74.4 (9.7, 58.2-92.2)[a]	75.7 (10.1, 55.7-89.6)[b]
Age<65, *n* (%)	21 (13.0)	14 (8.9)	15 (9.0)
Age<72.7, *n* (%)	116 (71.6)	64 (40.5)[a]	63 (37.7)[b]
Education, years, median (IQR, range)	16.0 (3.0, 12-20)	17.5 (4.3, 12-20)	16.0 (4.0, 12-20)[c]
APOE-ε4, *n* (%)	39 (24.7)	71 (47.0)[a]	106 (68.4)[b, c]
APOE-ε2, *n* (%)	19 (12.0)	6 (4.0)[a]	3 (1.9)[b]
KL-VS^{het+}, *n* (%)	43 (30.5)	39 (30.5)	20 (17.7)[b, c]
Aβ PET Centiloids, median (IQR, range)	7.1 (11.2, -17.3-22.4)	48.4 (45.4, 20.7-152.3)[a]	87.9 (52.3, 16.4-253.4)[b,c]
Participants with ≥2 Aβ PET scans (n = 199)			
A/T/N, *n* (%)	80 (40.2)	63 (31.7)	56 (28.1)
Diagnosis, CU:CI	80:0	45:18	24:32
Females, *n* (%)	53 (66.2)	28 (44.4)[a]	34 (60.7)
Age, years, median (IQR, range)	69.2 (8.7, 58.4-83.7)	76.2 (8.9, 62.2-91.5)[a]	76.2 (10.1, 56.3-90.4)[b]
Age<65, *n* (%)	8 (10.0)	3 (4.8)	5 (8.9)
Age<72.7, *n* (%)	54 (67.5)	21 (33.3)[a]	20 (35.7)[b]
Education, years, median (IQR, range)	17.5 (2.0, 11-20)	17.0 (3.5, 12-20)	16.0 (5.0, 12-20)[b,c]
APOE-ε4, *n* (%)	24 (30.4)	31 (49.2)	36 (64.3)[b]
APOE-ε2, *n* (%)	9 (11.4)	2 (3.2)	2 (3.6)
KL-VS^{het+}, *n* (%)	21 (28.4)	15 (25.0)	12 (21.8)
Aβ PET Centiloids, median (IQR, range)	6.8 (11.1, -8.3-22.4)	41.2 (35.4, 21.6-145.4)[a]	85.2 (56.8, 16.4-140.6)[b,c]
FU visits, median (IQR, range)	2 (0, 2-3)	2 (0, 2-3)	2 (0, 2-3)
Duration of FU, years, median (IQR, range)	2.1 (0.4, 1.3-4.2)	2.0 (0.2, 0.8-3.8)[a]	2.0 (0.3, 1.0-3.7)
Participants with ≥2 tau PET scans (n = 192)			

A/T/N, *n* (%)	37 (19.3)	80 (41.7)	75 (39.1)
Diagnosis, CU:CI	37:0	52:28	21:54
Females, *n* (%)	23 (62.2)	35 (43.8)	44 (58.7)
Age, years, median (IQR, range)	69.2 (9.3, 62.8-82.7)	74.4 (9.7, 58.2-92.2)[a]	75.7 (10.1, 55.7-89.6)[b]
Age<65, *n* (%)	6 (16.2)	5 (6.3)	9 (12.0)
Age<72.7, *n* (%)	26 (70.3)	32 (40.0)[a]	30 (40.0)[b]
Education, years, median (IQR, range)	16.0 (3.0, 12-20)	17.5 (4.3, 12-20)	16.0 (4.0, 12-20)[c]
APOE-ε4, *n* (%)	12 (32.4)	42 (53.8)	53 (74.6)[b,c]
APOE-ε2, *n* (%)	4 (10.8)	3 (3.9)	1 (1.4)
KL-VS^{het+}, *n* (%)	14 (41.2)	22 (31.9)	11 (18.6)
Aβ PET Centiloids, median (IQR, range)	5.0 (9.1, -6.4-22.4)	55.6 (39.4, 20.7-145.4)[a]	88.2 (52.0, 25.6-147.8)[b,c]
FU visits, median (IQR, range)	2 (1, 2-5)	2 (1, 2-4)	2 (1, 2-4)
Duration of FU, years, median (IQR, range)	2.0 (1.1, 0.9-3.7)	1.5 (1.0, 0.8-3.6)[a]	1.5 (1.1, 0.8-3.9)

A/T/N: amyloid-β/tau/neurodegeneration; CU: cognitive unimpaired; CI: cognitive impairment; IQR: interquartile range; FU: follow-up.

[a]: significant difference between the control and A+/T- at $p < 0.05$ with Benjamini-Hochberg corrected;

[b]: significant difference between the control and A+/T+ at $p < 0.05$ with Benjamini-Hochberg corrected;

[c]: significant difference between A+/T- and A+/T+ at $p < 0.05$ with Benjamini-Hochberg corrected.

Temporal meta-ROI FTP SUVR threshold (1.27)

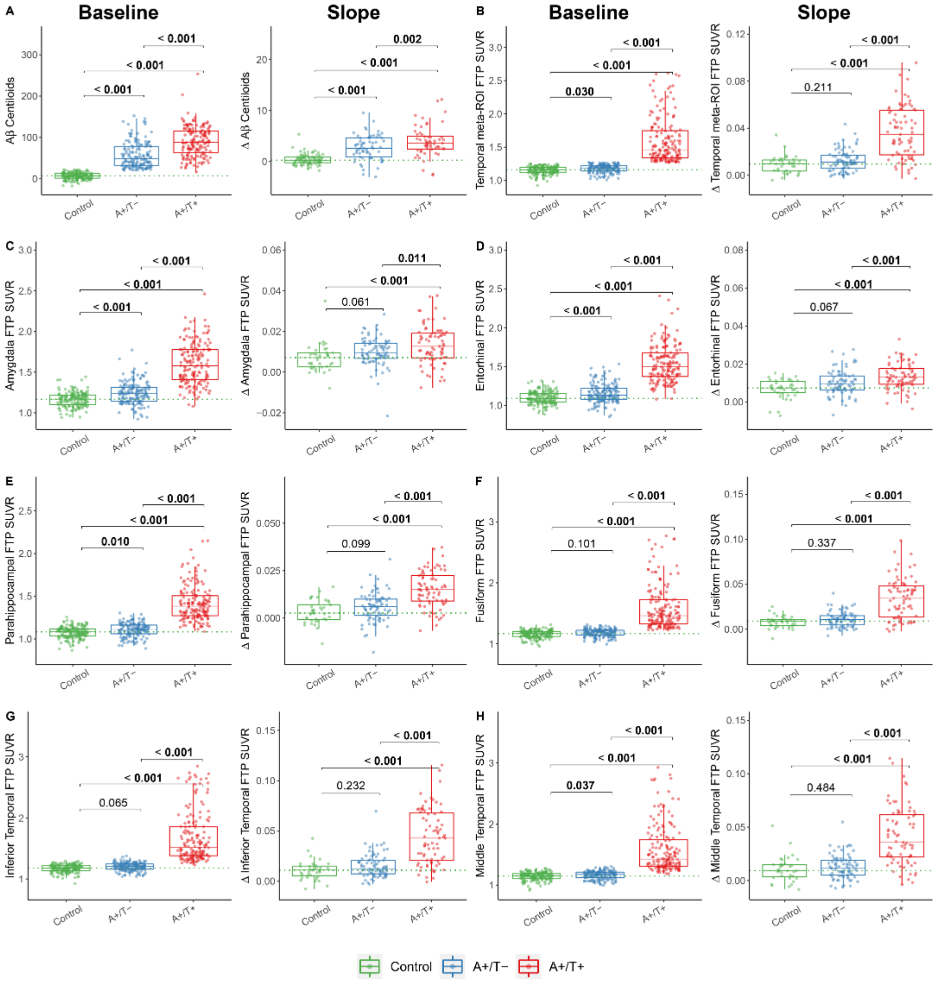

Figure S13. Comparisons of baseline and slopes of tau PET in temporal meta-ROI region and 6 ROIs in this composite region among the control, A+/T- and A+/T+ groups. Comparisons of baseline and slopes of **(A)** Aβ PET Centiloids, **(B)** tau PET in temporal meta-ROI region, **(C)** amygdala, **(D)** entorhinal, **(E)** parahippocampal, **(F)** fusiform, **(G)** inferior temporal and **(H)** middle temporal regions. The boxplot whiskers extended to the lowest and highest data points within 1.5 times the interquartile range from the lower to the upper quartiles. The control, A+/T- and A+/T+ individuals were colored in green, blue and red respectively. Green dashed lines represented the median value of the control group. $P <$ 0.05 were marked in bold.

Table S14. Comparisons of baseline and slopes of Aβ Centiloids, regional tau PET of temporal meta-ROI.

		Control vs. A+/T-	Control vs. A+/T+	A+/T- vs. A+/T+			Control vs. A+/T-	Control vs. A+/T+	A+/T- vs. A+/T+
Aβ	β_{std}	1.091	1.839	0.748	Parahip. tau	β_{std}	0.199	1.713	1.515
	95% ci	0.947~1.235	1.697~1.981	0.611~0.884		95% ci	0.049~0.349	1.566~1.861	1.373~1.657
	p	**< 0.001**	**< 0.001**	**< 0.001**		*p*	**0.010**	**< 0.001**	**< 0.001**
Δ Aβ	β_{std}	0.8675	1.336	0.469	Δ Parahip. tau	β_{std}	0.281	1.260	0.979
	95% ci	0.594~1.141	1.053~1.619	0.170~0.767		95% ci	-0.051~0.613	0.925~1.595	0.711~1.247
	p	**< 0.001**	**< 0.001**	**0.002**		*p*	0.099	**< 0.001**	**< 0.001**
Temporal meta-ROI tau	β_{std}	0.178	1.627	1.449	Fusi. tau	β_{std}	0.141	1.528	1.388
	95% ci	0.018~0.337	1.469~1.784	1.298~1.601		95% ci	-0.027~0.309	1.363~1.694	1.228~1.547
	p	**0.030**	**< 0.001**	**< 0.001**		*p*	0.101	**< 0.001**	**< 0.001**
Δ Temporal meta-ROI tau	β_{std}	0.194	1.419	1.224	Δ Fusi. tau	β_{std}	0.152	1.3562	1.2047
	95% ci	-0.109~0.498	1.112~1.725	0.979~1.470		95% ci	-0.157~0.460	1.044~1.668	0.955~1.454
	p	0.211	**< 0.001**	**< 0.001**		*p*	0.337	**< 0.001**	**< 0.001**
Amygd. tau	β_{std}	0.316	1.699	1.384	Inf-temp. tau	β_{std}	0.156	1.568	1.413
	95% ci	0.161~0.471	1.547~1.852	1.237~1.530		95% ci	-0.009~0.321	1.406~1.731	1.26~1.569
	p	**< 0.001**	**< 0.001**	**< 0.001**		*p*	0.065	**< 0.001**	**< 0.001**
Δ Amygd. tau	β_{std}	0.362	0.760	0.399	Δ Inf-temp. tau	β_{std}	0.188	1.383	1.195
	95% ci	-0.0141~0.737	0.381~1.140	0.095~0.703		95% ci	-0.120~0.497	1.072~1.695	0.9459 ~1.444
	p	0.061	**< 0.001**	**0.011**		*p*	0.232	**< 0.001**	**< 0.001**
Entorh. tau	β_{std}	0.258	1.749	1.491	Mid-temp. tau	β_{std}	0.181	1.536	1.355
	95% ci	0.110~0.405	1.603~1.894	1.351~1.631		95% ci	0.012~0.350	1.369~1.703	1.195~1.516
	p	**< 0.001**	**< 0.001**	**< 0.001**		*p*	**0.037**	**< 0.001**	**< 0.001**
Δ Entorh. tau	β_{std}	0.347	0.879	0.532	Δ Mid-temp. tau	β_{std}	0.1127	1.289	1.177
	95% ci	-0.022~0.716	0.506~1.251	0.234~0.830		95% ci	-0.202~0.428	0.971~1.608	0.922~1.431
	p	0.067	**< 0.001**	**< 0.001**		*p*	0.484	**< 0.001**	**< 0.001**

Δ: longitudinal tau increase; Amygd.: amygdala; Entorh.: entorhinal; Parahip.: parahippocampal; Fusi.: fusiform; Inf-temp.: inferior temporal; Mid-temp.: middle temporal.
$P < 0.05$ was indicated in bold.

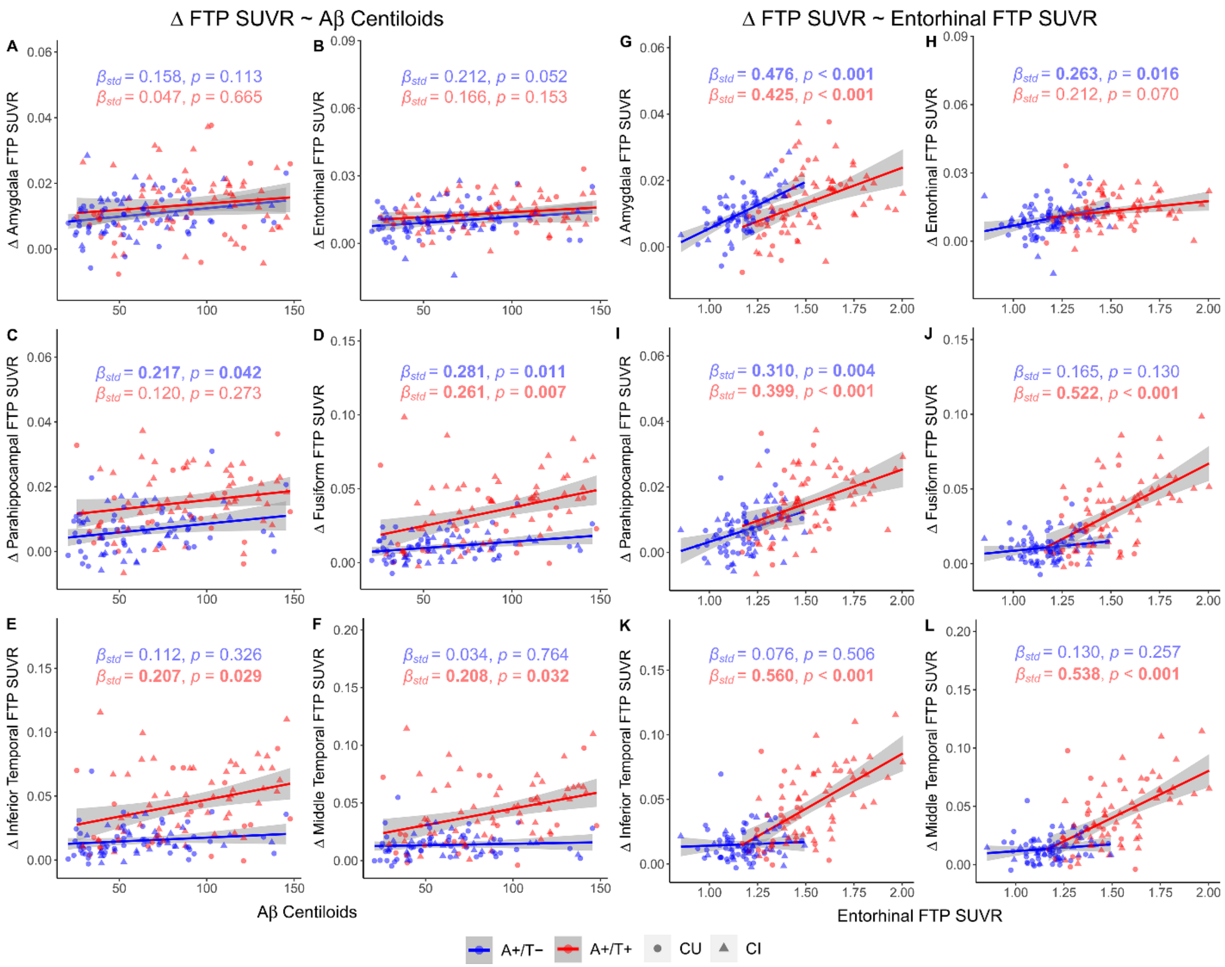

Figure S14. Prediction of longitudinal tau accumulations by baseline Aβ Centiloids and entorhinal tau. Models have been accounted for age and sex. Prediction of longitudinal FTP SUVR increases in **(A)** amygdala, **(B)** entorhinal, **(C)** parahippocampal, **(D)** fusiform, **(E)** inferior temporal and **(F)** middle temporal regions by baseline Aβ Centiloids. Prediction of longitudinal FTP SUVR increase in **(G)** amygdala, **(H)** entorhinal, **(I)** parahippocampal, **(J)** fusiform, **(K)** inferior temporal and **(L)** middle temporal regions by baseline entorhinal FTP SUVR. Linear regression lines, each individual point, β_{std} and p values of A+/T- and A+/T+ groups were colored in blue and red, and CU and CI individuals were represented by circle and triangle respectively. Linear model fits were indicated with 95% confidence intervals. $P < 0.05$ and associated β_{std} were marked in bold.

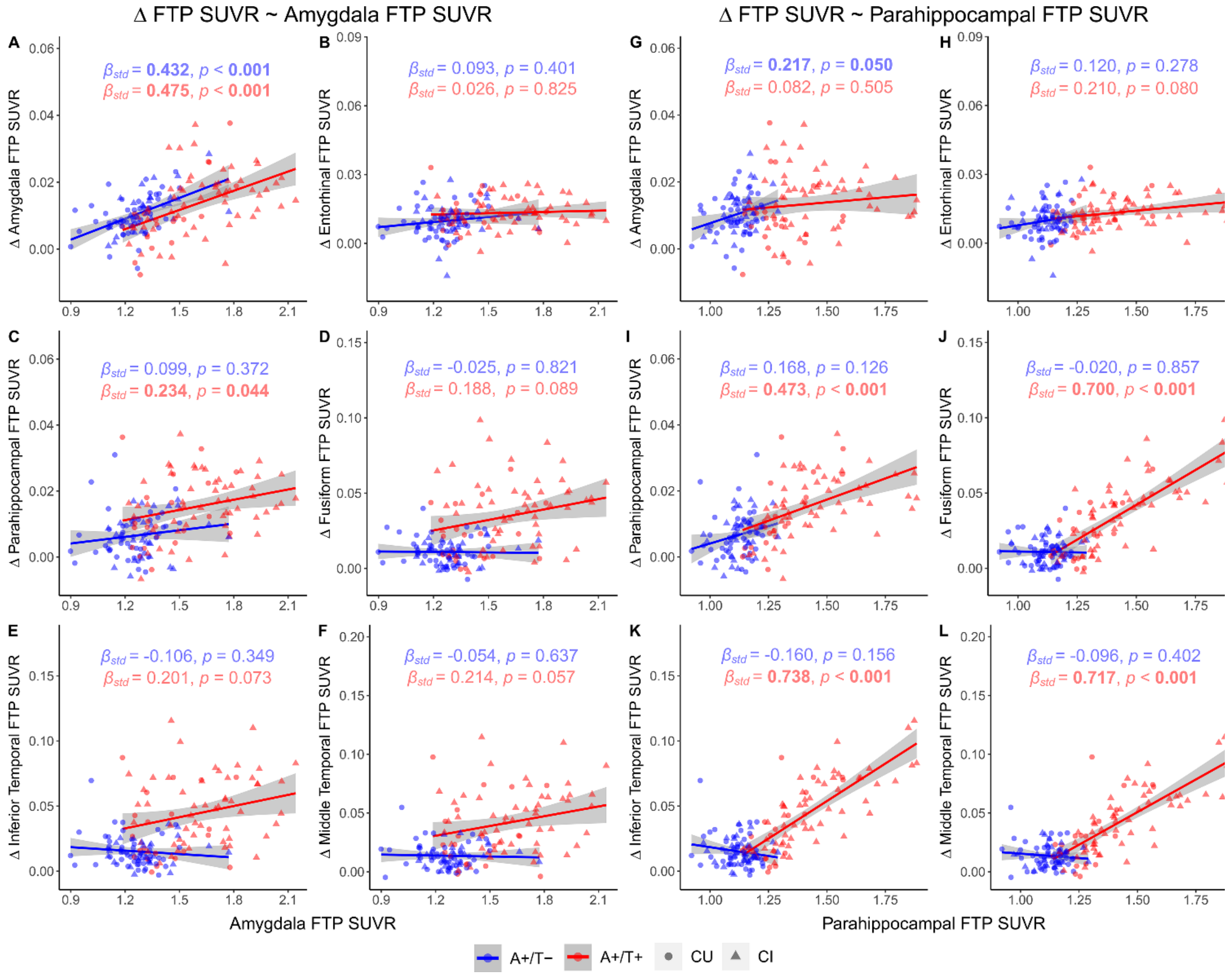

Figure S15. Prediction of longitudinal tau accumulations by baseline tau in amygdala or parahippocampal. Models have been accounted for age, sex and baseline Aβ. Prediction of longitudinal FTP SUVR increases in **(A)** amygdala, **(B)** entorhinal, **(C)** parahippocampal, **(D)** fusiform, **(E)** inferior temporal and **(F)** middle temporal regions by baseline amygdala FTP SUVR. Prediction of longitudinal FTP SUVR increases in **(G)** amygdala, **(H)** entorhinal, **(I)** parahippocampal, **(J)** fusiform, **(K)** inferior temporal and **(L)** middle temporal regions by baseline parahippocampal FTP SUVR. Linear regression lines, each individual point, β_{std} and p values of A+/T- and A+/T+ groups were colored in blue and red, and CU and CI individuals were represented by circle and triangle respectively. Linear model fits were indicated with 95% confidence intervals. $P < 0.05$ and associated β_{std} were marked in bold.

Table S15. Prediction of longitudinal tau accumulation by baseline amygdala tau or entorhinal tau adjusted for baseline Aβ Centiloids.

		Aβ + Amygd. tau				Aβ + Entorh. tau			
		A+/T-		A+/T+		A+/T-		A+/T+	
		Aβ	Amygd. tau	Aβ	Amygd. tau	Aβ	Entorh. tau	Aβ	Entorh. tau
Δ Amygd. tau	β_{std}	0.194	0.432	0.051	0.475	0.158	0.476	0.047	0.425
	95% ci	-0.002~0.390	0.235~0.628	-0.155~0.256	0.270~0.680	-0.035~0.350	0.283~0.668	-0.165~0.260	0.212~0.637
	p	0.056	**< 0.001**	0.631	**< 0.001**	0.113	**< 0.001**	0.665	**< 0.001**
Δ Entorh. tau	β_{std}	0.236	0.093	0.206	0.026	0.212	0.263	0.166	0.212
	95% ci	0.020~0.452	-0.123~0.309	-0.023~0.436	-0.204~0.256	0.002~0.422	0.053~0.473	-0.059~0.392	-0.014~0.438
	p	**0.036**	0.401	0.082	0.825	0.052	**0.016**	0.153	0.070
Δ Parahip. tau	β_{std}	0.246	0.099	0.161	0.234	0.217	0.310	0.120	0.399
	95% ci	0.030~0.461	-0.117~0.314	-0.062~0.385	0.011~0.458	0.011~0.423	0.104~0.517	-0.093~0.332	0.187~0.611
	p	**0.028**	0.372	0.162	**0.044**	**0.042**	**0.004**	0.273	**< 0.001**
Δ Fusi. tau	β_{std}	0.298	-0.025	0.337	0.188	0.281	0.165	0.261	0.522
	95% ci	0.085~0.511	-0.238~0.189	0.123~0.550	-0.026~0.402	0.070~0.492	-0.046~0.376	0.077~0.444	0.339~0.706
	p	**0.008**	0.821	**0.003**	0.089	**0.011**	0.130	**0.007**	**< 0.001**
Δ Inf-temp. tau	β_{std}	0.123	-0.106	0.288	0.201	0.112	0.076	0.207	0.560
	95% ci	-0.098~0.343	-0.327~0.115	0.071~0.506	-0.016~0.419	-0.110~0.334	-0.146~0.298	0.025~0.390	0.377~0.742
	p	0.280	0.349	**0.011**	0.073	0.326	0.506	**0.029**	**< 0.001**
Δ Mid-temp. tau	β_{std}	0.048	-0.054	0.282	0.214	0.034	0.130	0.208	0.538
	95% ci	-0.174~0.271	-0.277~0.169	0.065~0.499	-0.003~0.431	-0.188~0.257	-0.093~0.352	0.021~0.394	0.352~0.723
	p	0.671	0.637	**0.013**	0.057	0.764	0.257	**0.032**	**< 0.001**

Δ: longitudinal tau increase; Amygd.: amygdala; Entorh.: entorhinal; Parahip.: parahippocampal; Fusi.: fusiform; Inf-temp.: inferior temporal; Mid-temp.: middle temporal.
P < 0.05 was indicated in bold.

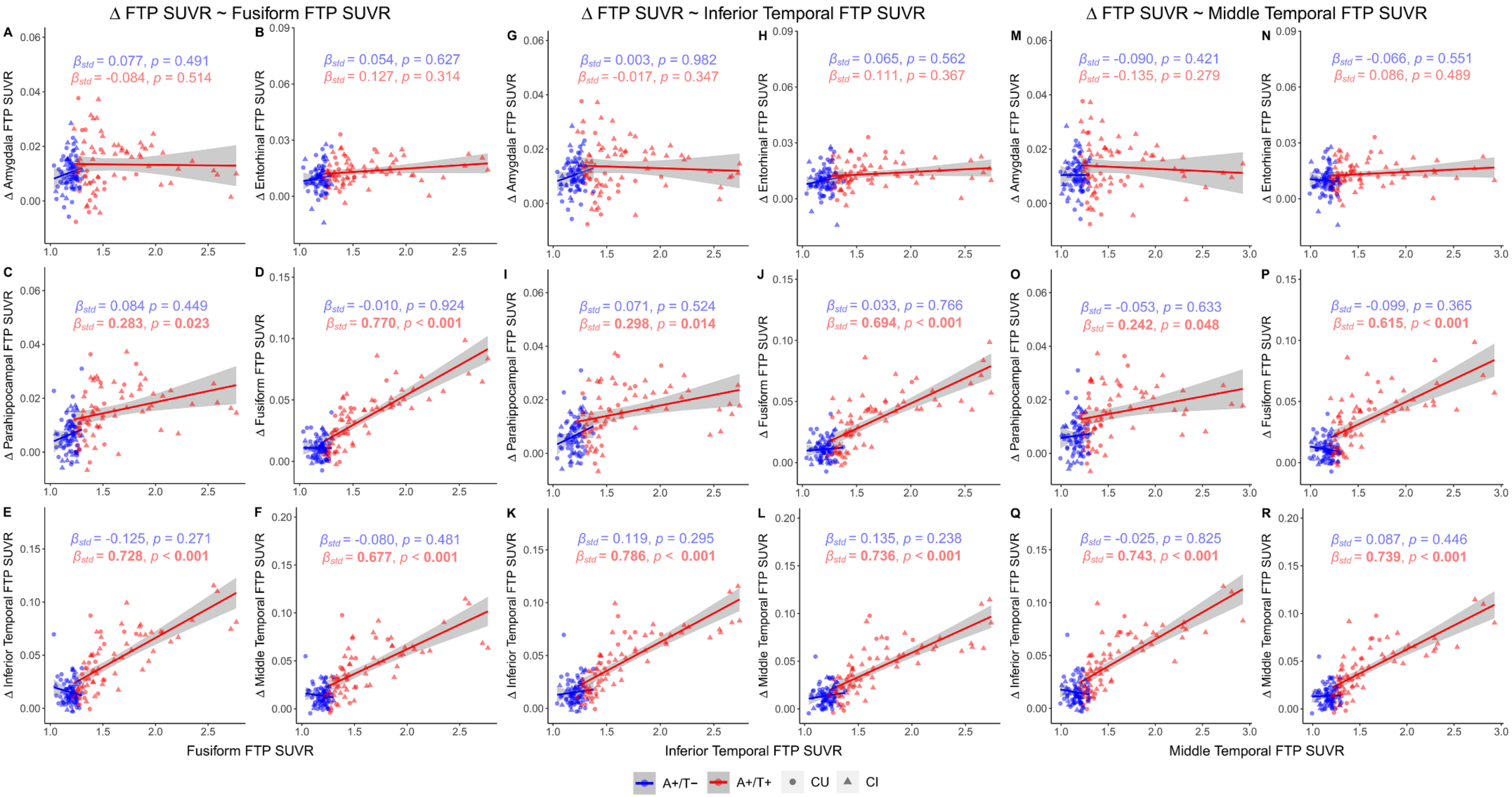

Figure S16. Prediction of longitudinal tau accumulation by baseline tau in fusiform, inferior temporal or middle temporal regions. Models have been accounted for age, sex and baseline Aβ. Prediction of longitudinal FTP SUVR increases in **(A)** amygdala, **(B)** entorhinal, **(C)** parahippocampal, **(D)** fusiform,

(E) inferior temporal and **(F)** middle temporal regions by baseline fusiform FTP SUVR. Prediction of longitudinal FTP SUVR increases in **(G)** amygdala, **(H)** entorhinal, **(I)** parahippocampal, **(J)** fusiform, **(K)** inferior temporal and **(L)** middle temporal regions by baseline inferior temporal FTP SUVR. Prediction of longitudinal FTP SUVR increases in **(M)** amygdala, **(N)** entorhinal, **(O)** parahippocampal, **(P)** fusiform, **(Q)** inferior temporal and **(R)** middle temporal regions by baseline middle temporal FTP SUVR. Linear regression lines, each individual point, β_{std} and p values of A+/T- and A+/T+ groups were colored in blue and red, and CU and CI individuals were represented by circle and triangle respectively. Linear model fits were indicated with 95% confidence intervals. $P < 0.05$ and associated β_{std} were marked in bold.

Table S16. Prediction of longitudinal tau accumulation by baseline parahippocampal tau or fusiform tau adjusted for Aβ Centiloids.

		Aβ + Parahip. tau				Aβ + Fusi. tau			
		A+/T-		A+/T+		A+/T-		A+/T+	
		Aβ	Parahip. tau	Aβ	Parahip. tau	Aβ	Fusi. tau	Aβ	Fusi. tau
Δ Amygd. tau	β_{std}	0.205	0.217	0.112	0.082	0.204	0.077	0.170	-0.084
	95% ci	-0.008~0.418	0.004~0.430	-0.127~0.351	-0.157~0.321	-0.014~0.422	-0.141~0.295	-0.080~0.420	-0.333~0.166
	p	0.063	**0.050**	0.362	0.505	0.071	0.491	0.186	0.514
Δ Entorh. tau	β_{std}	0.238	0.120	0.148	0.210	0.237	0.054	0.159	0.127
	95% ci	0.023~0.453	-0.095~0.335	-0.084~0.380	-0.022~0.442	0.020~0.454	-0.163~0.271	-0.086~0.405	-0.119~0.372
	p	**0.034**	0.278	0.215	0.080	**0.035**	0.627	0.208	0.314
Δ Parahip. tau	β_{std}	0.248	0.168	0.062	0.473	0.247	0.084	0.088	0.283
	95% ci	0.035~0.461	-0.045~0.382	-0.149~0.272	0.263~0.683	0.031~0.462	-0.132~0.299	-0.151~0.327	0.044~0.522
	p	**0.025**	0.126	0.567	**< 0.001**	**0.028**	0.449	0.471	**0.023**
Δ Fusi. tau	β_{std}	0.297	-0.020	0.161	0.700	0.298	-0.010	0.057	0.770
	95% ci	0.084~0.511	-0.233~0.194	0.004~0.317	0.544~0.856	0.084~0.511	-0.224~0.203	-0.097~0.210	0.617~0.924
	p	**0.008**	0.857	**0.048**	**< 0.001**	**0.008**	0.924	0.470	**< 0.001**
Δ Inf-temp. tau	β_{std}	0.120	-0.160	0.104	0.738	0.122	-0.125	0.028	0.728
	95% ci	-0.099~0.339	-0.379~0.059	-0.049~0.257	0.585~0.891	-0.098~0.342	-0.345~0.096	-0.142~0.199	0.558~0.898
	p	0.287	0.156	0.188	**< 0.001**	0.281	0.271	0.744	**< 0.001**
Δ Mid-temp. tau	β_{std}	0.047	-0.096	0.106	0.717	0.048	-0.080	0.045	0.677
	95% ci	-0.175~0.269	-0.318~0.127	-0.053~0.264	0.559~0.876	-0.174~0.271	-0.303~0.142	-0.137~0.227	0.495~0.858
	p	0.679	0.402	0.196	**< 0.001**	0.671	0.481	0.629	**< 0.001**

Δ: longitudinal tau increase; Amygd.: amygdala; Entorh.: entorhinal; Parahip.: parahippocampal; Fusi.: fusiform; Inf-temp.: inferior temporal; Mid-temp.: middle temporal.
$P < 0.05$ was indicated in bold.

Table S17. Prediction of longitudinal tau accumulation by baseline inferior temporal tau or middle temporal tau adjusted for Aβ Centiloids.

		Aβ + Inf-temp. tau				Aβ + Mid-temp. tau			
		A+/T-		A+/T+		A+/T-		A+/T+	
		Aβ	Inf-temp. tau	Aβ	Inf-temp. tau	Aβ	Mid-temp. tau	Aβ	Mid-temp. tau
Δ Amygd. tau	β_{std}	0.112	0.003	0.178	-0.117	0.201	-0.090	0.185	-0.135
	95% ci	-0.015~0.424	-0.217~0.222	-0.065~0.421	-0.360~0.126	-0.017~0.419	-0.308~0.128	-0.058~0.428	-0.378~0.108
	p	0.071	0.982	0.156	0.347	0.074	0.421	0.141	0.279
Δ Entorh. tau	β_{std}	0.232	0.065	0.172	0.111	0.235	-0.066	0.180	0.086
	95% ci	0.015~0.450	-0.153~0.282	-0.068~0.412	-0.129~0.351	0.018~0.452	-0.283~0.151	-0.061~0.421	-0.155~0.327
	p	**0.039**	0.562	0.164	0.367	**0.037**	0.551	0.147	0.489
Δ Parahip. tau	β_{std}	0.242	0.071	0.099	0.298	0.246	-0.053	0.117	0.242
	95% ci	0.026~0.459	-0.146~0.287	-0.133~0.330	0.066~0.529	0.030~0.462	-0.269~0.163	-0.119~0.352	0.007~0.478
	p	**0.031**	0.524	0.406	**0.014**	**0.029**	0.633	0.335	**0.048**
Δ Fusi. tau	β_{std}	0.295	0.033	0.127	0.694	0.293	-0.099	0.150	0.615
	95% ci	0.081~0.509	-0.181~0.247	-0.037~0.291	0.530~0.857	0.081~0.505	-0.311~0.114	-0.030~0.331	0.435~0.796
	p	**0.009**	0.766	0.135	**< 0.001**	**0.008**	0.365	0.107	**< 0.001**
Δ Inf-temp. tau	β_{std}	0.110	0.119	0.049	0.786	0.119	-0.025	0.059	0.743
	95% ci	-0.111~0.331	-0.102~0.340	-0.098~0.195	0.639~0.932	-0.103~0.341	-0.247~0.197	-0.100~0.218	0.584~0.902
	p	0.333	0.295	0.518	**< 0.001**	0.298	0.825	0.468	**< 0.001**
Δ Mid-temp. tau	β_{std}	0.036	0.135	0.062	0.736	0.051	0.087	0.056	0.739
	95% ci	-0.186~0.258	-0.087~0.357	-0.099~0.222	0.575~0.896	-0.172~0.273	-0.136~0.309	-0.104~0.216	0.579~0.900
	p	0.753	0.238	0.453	**< 0.001**	0.656	0.446	0.496	**< 0.001**

Δ: longitudinal tau increase; Amygd.: amygdala; Entorh.: entorhinal; Parahip.: parahippocampal; Fusi.: fusiform; Inf-temp.: inferior temporal; Mid-temp.: middle temporal.
P < 0.05 was indicated in bold.

References

1. Desikan RS, Ségonne F, Fischl B, Quinn BT, Dickerson BC, Blacker D, et al. An automated labeling system for subdividing the human cerebral cortex on MRI scans into gyral based regions of interest. Neuroimage. 2006;31(3):968–80.
2. Landau SM, Fero A, Baker SL, Koeppe R, Mintun M, Chen K, et al. Measurement of Longitudinal -Amyloid Change with 18F-Florbetapir PET and Standardized Uptake Value Ratios. J Nucl Med. 2015;56(4):567–74.
3. Guo T, Landau SM, Jagust WJ. Age, vascular disease, and Alzheimer's disease pathologies in amyloid negative elderly adults. Alzheimers Res Ther. 2021 Dec;13(1):174.
4. Jack CR, Wiste HJ, Weigand SD, Therneau TM, Lowe VJ, Knopman DS, et al. Defining imaging biomarker cut points for brain aging and Alzheimer's disease. Alzheimer's Dement. 2017 Mar;13(3):205–16.
5. Harrison TM, La Joie R, Maass A, Baker SL, Swinnerton K, Fenton L, et al. Longitudinal tau accumulation and atrophy in aging and alzheimer disease. Ann Neurol. 2019 Feb;85(2):229–40.
6. Hanseeuw BJ, Betensky RA, Jacobs HIL, Schultz AP, Sepulcre J, Becker JA, et al. Association of Amyloid and Tau With Cognition in Preclinical Alzheimer Disease. JAMA Neurol. 2019 Aug;76(8):915.
7. Guo T, Korman D, La Joie R, Shaw LM, Trojanowski JQ, Jagust WJ, et al. Normalization of CSF pTau measurement by Aβ40 improves its performance as a biomarker of Alzheimer's disease. Alzheimers Res Ther. 2020;12(1):97.
8. Jack CR, Bernstein MA, Fox NC, Thompson P, Alexander G, Harvey D, et al. The Alzheimer's Disease Neuroimaging Initiative (ADNI): MRI methods. J Magn Reson Imaging. 2008;27(4):685–91.
9. Jack CR, Knopman DS, Weigand SD, Wiste HJ, Vemuri P, Lowe V, et al. An operational approach to National Institute on Aging-Alzheimer's Association criteria for preclinical Alzheimer disease. Ann Neurol. 2012;71(6):765–75.
10. Guo T, Korman D, Baker SL, Landau SM, Jagust WJ. Longitudinal Cognitive and Biomarker Measurements Support a Unidirectional Pathway in Alzheimer's Disease Pathophysiology. Biol Psychiatry. 2021 Apr;89(8):786–94.
